# Integrative analyses of metabolome and transcriptome reveals metabolomic variations and candidate genes involved in sweet cherry (*Prunus avium* L.) fruit quality during development and ripening

**DOI:** 10.1371/journal.pone.0260004

**Published:** 2021-11-15

**Authors:** Haiying Yang, Changping Tian, Shujun Ji, Fengzhu Ni, Xinguang Fan, Yanqing Yang, Chanchan Sun, Hansheng Gong, Aidi Zhang

**Affiliations:** 1 School of Food Engineering, Ludong University, Yantai, Shandong, 264025, PR China; 2 Cherry Research Department, Yantai Agricultural Science and Technology Institute, No.26, West Gangcheng Street, Yantai, 265500, China; 3 Key Laboratory of Food Nutrition and Safety, Tianjin University of Science and Technology, Ministry of Education, Tianjin, 300457, China; Bangabandhu Sheikh Mujibur Rahman Agricultural University, BANGLADESH

## Abstract

Sweet cherry (*Prunus avium* L.), one of the most appreciated and most important commercial temperate fruits, has high sensory quality and nutritional value. Investigating its metabolic variations provides valuable information on the formation of fruit quality. In this study, widely targeted LC-MS/MS based metabolomics was used to identify and quantify metabolic changes during ‘Black Pearl’ sweet cherry development and ripening. A total of 263 significant differentially expressed metabolites (DEMs) were detected during the four fruit-development stages. Significant differences were observed in the composition and content of compounds in the four stages of cherry development, especially sugars, organic acids, and flavonoids. Moreover, transcriptome analysis provided a molecular basis for metabolic variations during fruit development. A total of 6724 significant differentially expressed genes (DEGs) were identified. Further correlation analysis of major DEMs and DEGs showed that 19 key DEGs were involved in sugar metabolism, 23 key DEGs in organic acid metabolism, and 13 key DEGs in flavonoid metabolism. The upregulated genes involved in the flavonoid pathway probably play an important role in regulating the rapid increase of anthocyanin content during fruit development. These comprehensive analysis data provide a better understanding to improve fruit quality traits based on molecular and metabolic levels.

## Introduction

Sweet cherry (*Prunus avium* L.) is a popular commercial fruit crop because of its attractive appearance, delicious taste, and rich nutritional value [1,2]. These quality traits determine that it is mainly consumed as fresh fruit. Sweet cherries are also used to make jam, wine, fruit juice, dried fruit, candy, and other foods as high-quality ingredients [1,3]. Sweet cherry is an ideal model for fruit quality research due to the rapid changes in sugars, organic acids, pigments, aromatic volatile, and other quality components during development and ripening [2,4,5]. The accumulation of sugars and acids results in the unique taste, and they are essential factors for evaluating the physiological and sensory aspects of the fruit [5]. Due to various polyphenol chemicals (e.g., hydroxycinnamic acid, anthocyanins, and flavonols), sweet cherry has high antioxidant activity, which contributes to human health [1,6]. Therefore, studies on sweet cherry development and maturation processes can help us to understand changes in fruit quality and formation, especially particular properties associated with taste, color, and health-associated nutrients.

Taste, color, and flavor of foods are the essential factor that primarily determines the acceptability of foods [7,8]. Taste makes great contributions to fruit flavor [9], and is generally categorized as sweet or sour, both of which are determined by the composition and content of sugars and organic acids [10]. Sugars are not only the key substances that determine fruit sweetness but also nutritional and energy sources for the human body [9,11]. Organic acids contribute to the formation of the flavor and pH value of fruits and affect their sensory quality [12]. Fruit taste and maturity can be assessed by the ratio of sweetness and acidity [10]. At the same time, the taste is also affected by the abundance and composition of amino acids such as alanine, glutamate, aspartate, asparagine, and so on [13,14]. Sweet cherry fruit accumulates sugars with cell expansion, and the levels of soluble sugar, especially glucose (Glc), increase rapidly during the degreening (DG) and full red (FR) stages [4]. Meanwhile, the content of organic acids decreases during fruit development, which could be attributed to the massive growth of fruit cells [15].

Additionally, plant secondary metabolites, particularly polyphenolic compounds, are thought to be very important and necessary for daily human consumption because of antioxidant activity [16]. Polyphenolic compounds can have beneficial effects on health, such as preventing cancer, controlling cardiovascular disease, modulating diabetes and inflammatory [17]. Phenolic compounds detected in red fruits of strawberry include anthocyanins, flavonols, flavan-3-ols, and others [18]. Twenty polyphenolic compounds were identified in apple; quercetin glycoside is the main ingredient in peel and phenolic acid is the main ingredient in flesh [19]. In kiwifruit, combined metabolome and transcriptome analysis revealed that flavonoid biosynthesis presented significant differences during *A*. *arguta* fruit development in red-fleshed and green-fleshed cultivars [20]. Sweet cherry contains high amounts of phenols, although various levels exist among different varieties [21]. Among them, anthocyanins, which are some of the most common phenolic compounds, not only have potent antioxidant activity but also contribute the most to the formation of red-purple colors [21–24]. In sweet cherry, anthocyanin accumulates during fruit development, and the cultivar with dark skin and flesh was shown to exhibit the highest anthocyanin content compared to red and yellow cultivars [25]. In our recent work, the anthocyanin content of sweet cherry was higher in the dark red stage than the initial red stage, and it largely accumulated in peel more than flesh at each stage [26]. These results confirmed that anthocyanin accumulation is the decisive factor for sweet cherry red coloration.

The main goal of metabolomics is to identify and quantitatively study dynamic metabolites in living organisms responding to external stimuli, pathophysiological changes, and their own genetic mutations [27]. Widely targeted metabolomics have been used to identify total metabolites and their qualitative and quantitative (relative) information at the maximum extent in biological samples including apple [9], peach [28], strawberry [29], and watermelon [30]. As we know, transcriptome sequencing technology has been widely employed for the detection of genes and markers in fruit development [31]. In recent years, the combined metabolomics/transcriptomic approach has gained attention because of its ability to effectively identify genes associated with metabolites [28]. Xu et al. [9] used the combined approach to examine the metabolites related to fruit quality during apple development and ripening. Gabay et al. [32] revealed the key role of α-linolenic acid and genes in the regulation of dormancy in pear by combined analysis. Flavonoid biosynthesis has been studied in kiwifruit through combined analysis of the fruit metabolome and transcriptome [20].

In the present study, we aimed to gain novel insights on the fruit quality formation of ‘Black Pearl’ sweet cherries by using the widely targeted metabolomics approach. Moreover, a combined analysis of metabolomics and transcriptomic was performed to study the correlation between significant differentially expressed metabolites (DEMs) and differentially expressed genes (DEGs) during sweet cherry development and ripening processes. To further investigate the quality formation, we focused on the DEMs and DEGs related to sugar, organic acid, and flavonoid metabolism. The comprehensive metabolic analysis in this study provided new insight to understand fruit quality formation based on metabolite changes and molecular mechanisms.

## Materials and methods

### Plant material

The sweet cherry (*Prunus avium* L. cv. ‘Black Pearl’) used in this study was harvested in 2019 from Yantai Agricultural Science and Technology Institute, Shandong, China (latitude: 37.4893, lngitude:121.2790; elevation: 10.2 m). Sweet cherry fruits of uniform size and without visible defects at different developmental stages (10, 25, 33, and 40 days after full bloom (DAFB)) were adopted as the experimental materials. Each stage had 3 biological replicates comprising 30 individual fruits, and each replicate comprised 10 fruits. The textural, sensory, and physiological properties were evaluated immediately after harvest. Then, the fruit flesh with peel removed was sliced into small pieces, transferred immediately to liquid nitrogen, and placed at -80°C for storage. All fruit samples were submitted for subsequent metabolome and transcriptome analyses.

### Fruit physiological properties

#### Determination of firmness

The firmness of sweet cherries was measured using a CT3 10K texture analyzer (AMETEK Brookfield, Middleboro, MA, USA) equipped with a 2 mm diameter cylinder probe. The pressed distance of the probe was 3 mm. The pressed speed of the probe was 2.3 mm/s. The trigger force used was 0.05 N. Firmness was recorded as peak force (N). Each stage had 3 biological replicates, and each replicate measured 10 sweet cherries.

#### Measurement of total soluble solids (TSS)

TSS was determined by an Atago digital hand-held refractometer (Japan). Three drops of juice were squeezed from each fruit onto the refractometer. Each stage had 3 biological replicates, with 10 cherries per replicate.

#### Measurement of skin and fruit color

Skin color change during sweet cherry development stages was measured with a HunterLab chromameter (Konica Minolta, Inc., Japan) by the L*a*b* color space (Commission International de l’Eclairage, CIE). Measurements were conducted on 2 flat surfaces of each fruit. Each stage had 3 biological replicates, with 10 fruits per replicate.

#### Determination of anthocyanin

Small pieces of sweet cherry were quickly ground to powder under liquid nitrogen. Approximately, 0.5 g of powder was transferred to a 10 mL vial and 2.5 mL of 0.01% HCL-methanol solution was immediately added before extraction at 4°C for 24 h in the dark. The supernatant was retrieved after samples were centrifuged at 12,000 rpm for 20 min. Then, 2 dilutions were used to dilute the samples separately: one consisting of 0.4 M potassium chloride buffer (pH 1.0) and one of 0.4 M dibasic sodium phosphate buffer (pH 4.5). To measure the absorbance, a UV-723N spectrophotometer (Youke, Shanghai, China) was applied to each sample at 510 and 700 nm. Total anthocyanin content was measured by the pH differential method, which can be calculated by the formula: A = [(A510 –A700) _pH 1.0_ –(A510 –A700) _pH 4.5_]. The results are shown as mg cyanidin-3-galactoside equivalent per 100 g fresh weight (FW). Three independent biological replicates were analyzed.

### Metabolite profiling analysis

#### Metabolite extraction

The freeze-dried samples were mashed with liquid nitrogen, and 50 mg of powder for each sample was transferred into a 1.5 mL EP tube containing 5 mL precooled methanol/water (3:1, v/v) and subjected to vigorous vortexing. Each sample point had 3 independent biological replicates. All samples were extracted at 4°C overnight. Then, the samples were centrifuged at 13,000 rpm for 15 min at 4°C and the filtered supernatant (0.22 μm membrane filter) was blow-dried with nitrogen. The samples were redissolved with 50 μL isopropanol/acetonitrile/water (2:7:1, v/v/v) and extracted by centrifugation before LC-MS/MS analysis.

#### LC-MS/MS analysis

The extracted samples of cherry were separated on the UPLC system (Dionex UltiMate 3000 RSLC) equipped with Waters ACQUITY UPLC CSH C18 column (130Å, 1.7 μm, 2.1 mm × 100 mm). The analysis conditions were as follows: column temperature, 45°C; injection volume, 1.5 μL; flow rate, 0.3 mL/min. The mobile phases were water (0.1% formic acid) (phase A) and acetonitrile (0.1% formic acid) (phase B). The gradient program of phase A/phase B was 98:2 (v/v) at 0 min, 98:2 (v/v) at 0.5 min, 2:98 (v/v) at 15 min, 2:98 (v/v) at 17 min, 2:98 (v/v), at 17.1 min and 98:2 (v/v) at 20 min.

Samples were inserted into quality control (QC) samples in queue mode to monitor and evaluate the stability of the system and the reliability of the experimental data. Each sample was operated in both positive and negative ion modes by the electrospray ionization (ESI) source parameters. Mass spectrometry was carried out by the Q-Exactive spectrometer (Thermo Scientific, San Jose, CA, USA) after the sample was separated by UPLC. The MS conditions were as follows: mass range (m/z) 80–1200; aux gas flow rate, 15 arb; sheath gas flow rate, 45 arb; source temperature, 320°C; spray voltage, 3.5 kV in positive mode and 3.2 kV in negative mode.

### Analysis of metabolomics data

After the original data were adjusted for peak alignment, peak area extraction, retention time correction and feature extraction using the Compound Discoverer 3.0 program, the metabolite structure was identified by accurate mass MS matching (< 25 ppm), and the MS1 and MS2 matching search MZcloud and ChemSpider database were used to determine the metabolite structure. For MZcloud database, the metabolites were identified by exact mass (m/z), molecular formula, and fragmentation spectrum (MS2). LC-MS/MS Mass chromatogram of ten representative metabolites were displayed in S1 File. The relative content of metabolites was expressed by peak area. After preprocessed by pattern recognition and Pareto-scaling, the peak area data for all metabolites were further analyzed by SIMCA-P 14.1 software (Umetrics, Umea, Sweden), including unsupervised principal component analysis (PCA), supervised partial least squares discriminant analysis (PLS-DA), and orthogonal partial least squares discriminant analysis (OPLS-DA). The quality of the PCA, PLS-DA, and OPLS-DA models was evaluated based on their *R*^2^*X* or *R*^2^*Y* and *Q*^2^ values (*R*^2^: interpretation rate of the model, *Q*^2^: predictability of the model). Metabolites with significant differences were screened with variable importance in projection (VIP) > 1 and an independent sample t-test (*p* < 0.05). Metabolite profiling and metabolomics data analyses were executed by Personal Biotechnology Cp. Ltd. (Shanghai, China).

### RNA-seq analysis

Total RNA was isolated from frozen sweet cherry flesh using an RNAprep Pure Plant Plus Kit (TIANGEN, Beijing, China) according to its instructions. RNA-seq and bioinformatics analyses were executed by Personal Biotechnology Cp. Ltd. (Shanghai, China). The sequencing libraries of samples were constructed by a TruSeq RNA Sample Preparation Kit (Illumina, San Diego, CA, USA), and the libraries were sequenced on the Illumina Hiseq X platform. The reference genome database was extracted from Genome Database for Rosaceae (https://www.rosaceae.org/species/prunus/all), and gene annotations were acquired from the genome. The index of the reference genome was built with Bowtie2 (2.2.6), and the clean reads were mapped to the reference genome using Tophat (2.0.14). HTSeq (0.9.1) was applied to the expression and quantification levels of genes to calculate fragments per kilobase per million fragments (FPKM). Then, the DEG were identified by DESeq (1.30.0) with screening conditions as follows: expression difference multiple |log_2_FoldChange| > 1 and significant *P*-value < 0.05. Subsequently, clustering analysis of all differential genes was performed using the R language Pheatmap (1.0.8) software package. Each point had three biological replicates for RNA-seq analysis. The raw data of RNA-seq were submitted under BioProject accession number PRJNA746102.

### Correlation analysis of transcriptomic and metabolomic data

A nine-quadrant graph was constructed to illustrate the fold change of DEMs and DEGs in each group, with |r| (correlation coefficient) > 0.8 among different groups, and the r between DEMs and DEGs was assessed by the cor function in the R package (https://cran.r-project.org/). To further observe the changes and associations of metabolites and genes, the top 10 significantly different metabolites (VIP > 1, |log_2_FoldChange| > 1, and *p* < 0.05) and significantly different genes (|log_2_FoldChange| > 1, *p* < 0.05 and FPKM > 10) in each of the four comparisons (10 vs. 25 DAFB, 25 vs. 33 DAFB, 33 vs. 40 DAFB, 10 vs. 40 DAFB) were used to describe a correlation network diagram with |r| > 0.90. The correlation analysis and network plot were performed with the OmicStudio tools at https://www.omicstudio.cn/tool.

### Statistical analysis

The statistical significance of the populations was calculated with Tukey’s test, and significance was indicated with different letters above the error bars. The heatmap was drawn by the TBtools software. The Venn diagram was displayed by OmicStudio tools (https://www.omicstudio.cn/tool). Figures were drawn by Origin 8.0 (OriginLab Corp., Northampton, MA, USA).

## Results

### Physiological indices during sweet cherry fruit development

**Fig 1:**
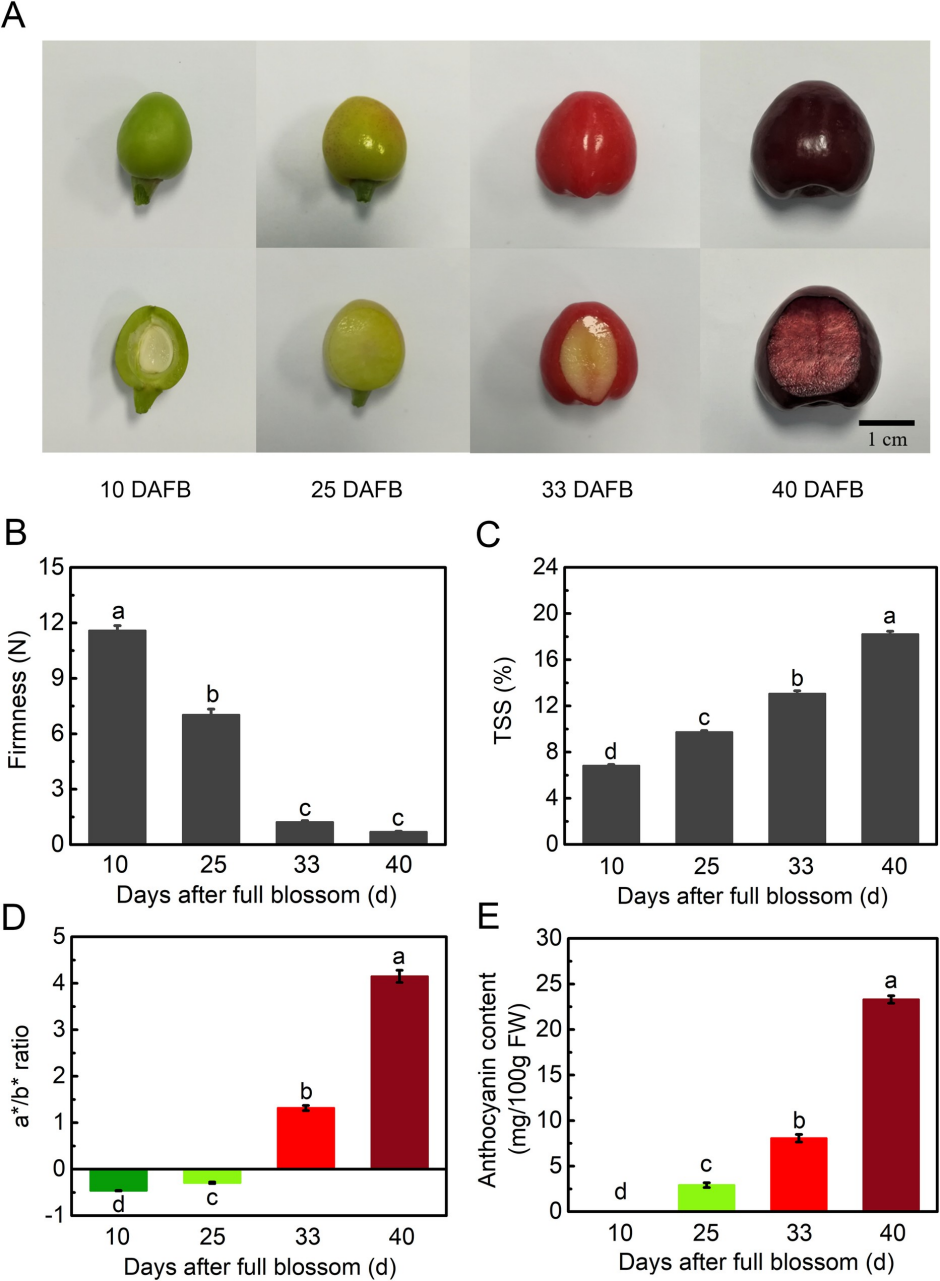
Physiological and morphological changes during sweet cherry (*Prunus avium* L. cv. ‘Black Pearl’) fruit development. (A) Photos of developmental stages. (B) Fruit firmness (N). (C) Changes in total soluble solids (TSS). (D) Changes in a*/b* ratio. Error bars in (B), (C), (D) represent ± SE from 10 replicates. (E) Anthocyanin content at different developmental stages. Error bars represent ± SE from three replicates. Tukey’s test significant at *p* < 0.05, indicated by a, b, c, and d.

To investigate quality and metabolic changes during the development of sweet cherry fruit, we chose four developmental stages (10, 25, 33, and 40 DAFB) for extensive experimentation and metabolic analysis. The appearance of sweet cherry fruit collected from different stages is shown in Fig 1A. Fruit color gradually turned to red and transformed to dark red at 40 DAFB (Fig 1A). Distinct physiological and morphological changes were observed at all development stages. Notably, fruit firmness decreased rapidly from 11.6 N at 10 DAFB to 1.2 N at 33 DAFB, and reached to 0.7 N at the dark red maturity stage at 40 DAFB (Fig 1B). The TSS was negatively correlated with firmness, increasing continually from 6.8% at 10 DAFB to 18.2% at 40 DAFB (Fig 1C). The a*/b* ratio was considered as a sign of fruit color, and in this work, it increased rapidly from -0.46 at 10 DAFB to 4.1 at 40 DAFB, indicating that the fruit color varied from green to red (Fig 1D). In addition, anthocyanin content was also consistent with color change during sweet cherry fruit development, reaching 23.3 mg/100 g FW at the dark red mature stage (Fig 1E).

### Metabolomic analysis of sweet cherry reveals dynamic metabolic profile change during developmental stages

**Fig 2:**
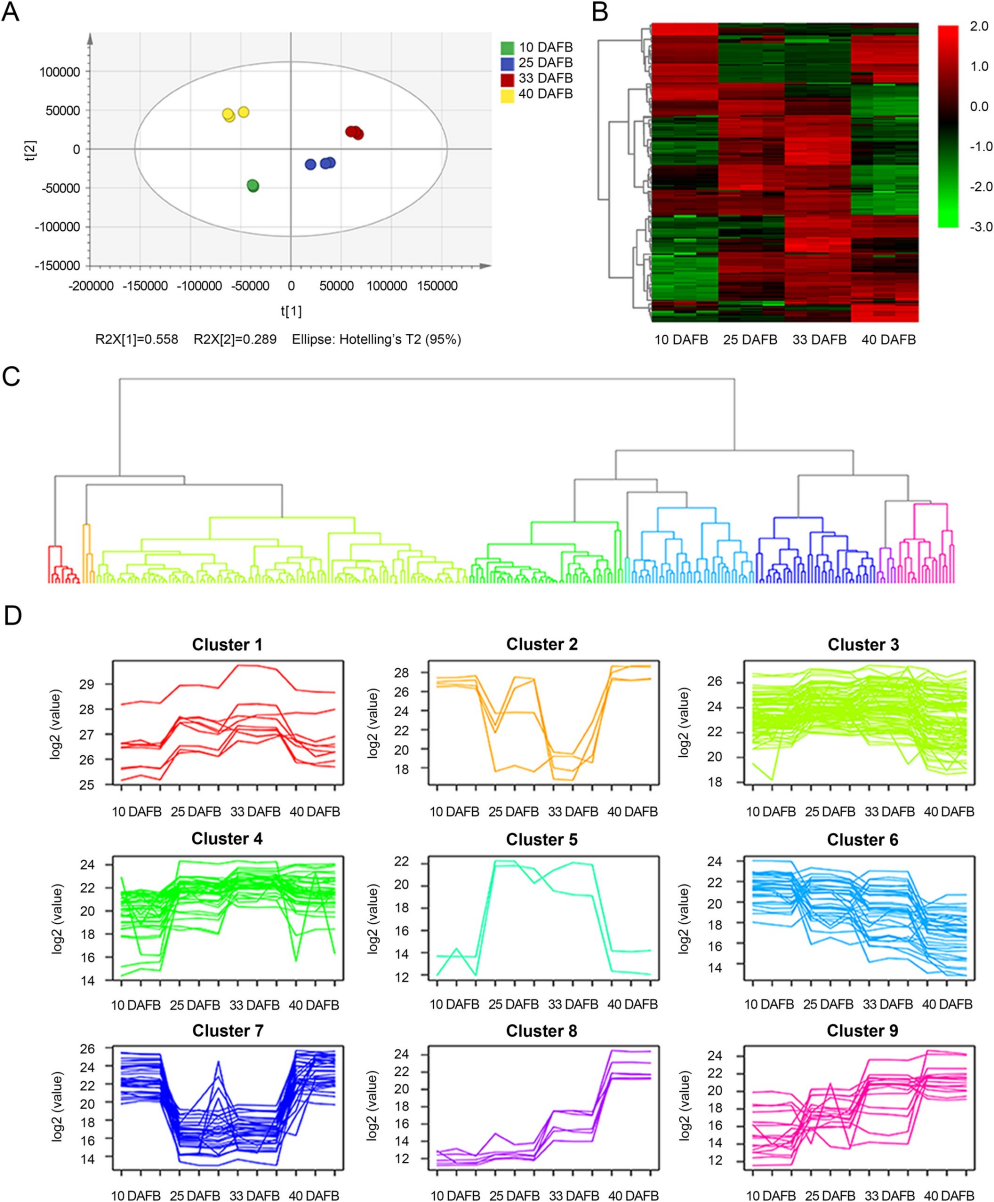
Dynamic metabolome of sweet cherry development and ripening. (A) Principal component analysis (PCA) of metabolites identified in developmental stages. (B) Heatmap of all differentially expressed metabolites (DEMs) at four developmental stages. Color indicates level of relative content of each DEM, from green (low) to red (high). (C) Clustering analysis of all DEMs according to their variation tendency, and (D) metabolite variation tendencies among the 9 cluster profiles.

To better understand the metabolic changes during the sweet cherry development process, widely targeted LC-MS/MS based metabolomics was applied during the four developmental stages. As shown in Fig 2A, PCA of the metabolomic profiles was conducted to provide information about distinctly separate groups among the samples of the four fruit-development stages and the three replicates of each stage together. The results indicate that the metabolites presented distinct variations at different stages and high reproducibility among replicates. Metabolic profiles in all developmental stages were also clearly distinguished in the PLS-DA score plots (S1 Fig). The OPLS-DA analysis indicated that they had satisfactory predictive capabilities (S1 Fig). The peak area data were used for PCA, PLS-DA, as well as OPLS-DA analysis. DEMs with significant differences were screened with VIP > 1 and *p*-value < 0.05. A total of 263 DEMs were identified among the four stages (S2 File). A heatmap with hierarchical clustering analysis of proportional content for all DEMs is shown in Fig 2B, and the results show that many metabolites were highly expressed at each development stage. About a third of DEMs were accumulating during fruit development (Fig 2B). Around one in five DEMs expressed high in green fruit stage (10 DAFB) and dark red stage (40 DAFB), but keep low level in degreening (25 DAFB) and full red phases (33 DAFB) (Fig 2B). There were also around one in five DEMs exhibited opposite trend (Fig 2B). The sugar content increased and the organic acid showed a slight rising trend accompanying with fruit ripening (S3 File). As for the secondary metabolites, flavonoids had the largest increase from green fruit to dark red fruit, while the phenylpropanoids, terpenoids and alkaloids showed an upregulation trend and then declining during the whole development period (S3 File). According to the variations of DEMs among the developmental stages, clustering analysis was carried out and DEMs were grouped into nine clusters according to their variation tendency (Fig 2C and 2D). The expression level of metabolites from clusters 8 and 9 showed similar growth trends in different developmental stages. Interestingly, this trend was also observed in TSS, a*/b* ratio, and anthocyanin content (Fig 1C–1E), which were closely related to the nutrition and color formation of sweet cherry fruits. Moreover, the flavonoids, including cyanidin, were mainly clustered in the cluster 8 profile (S2 File).

### Multivariate analysis of identified metabolites

**Fig 3:**
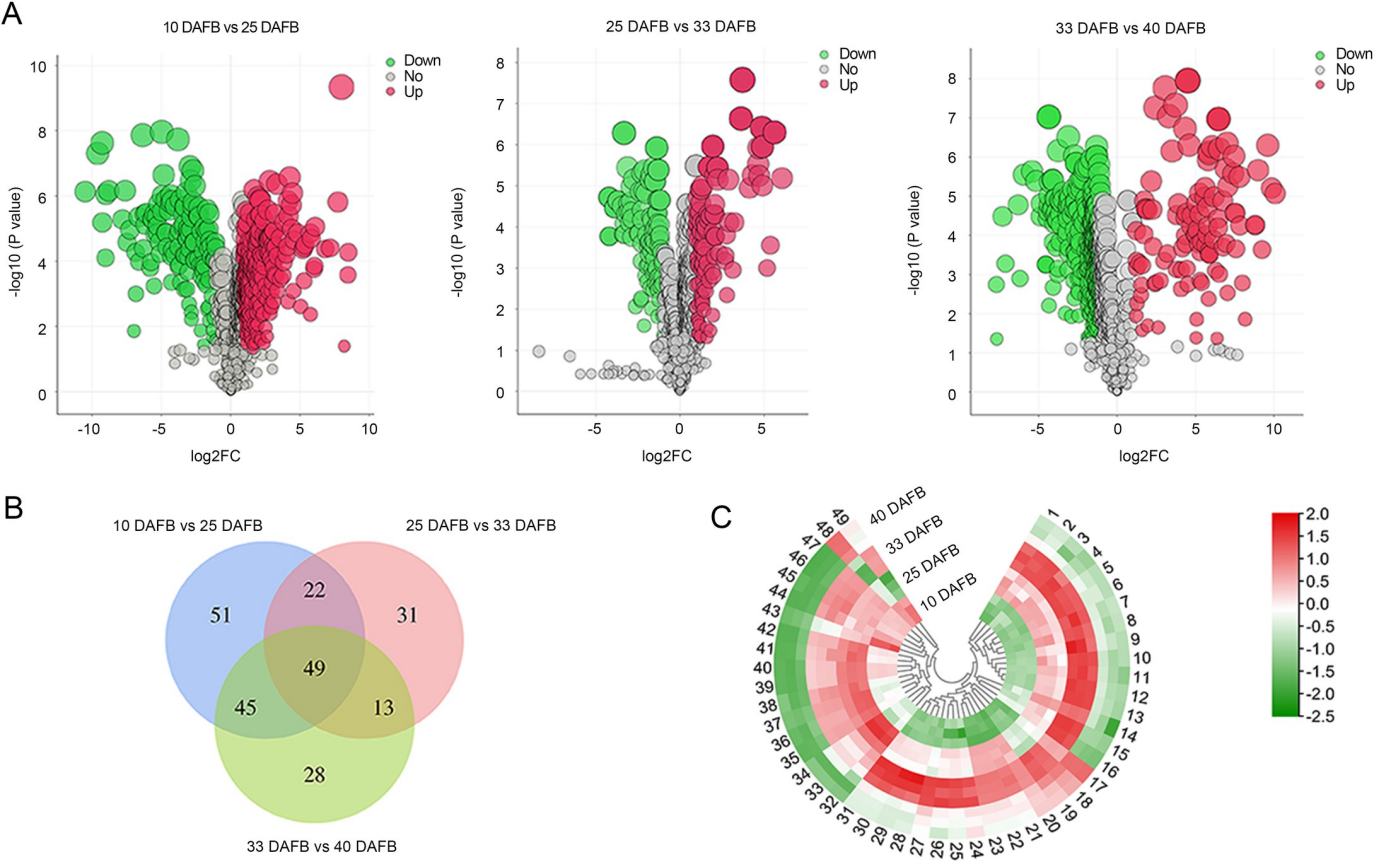
Differentially accumulated metabolites in sweet cherry at different fruit development stages. (A) Volcano plots of differential metabolites in 10 vs. 25 DAFB, 25 vs. 33 DAFB, and 33 vs. 40 DAFB, respectively. (B) Venn diagram of DEMs in 10 vs. 25 DAFB, 25 vs. 33 DAFB, and 33 vs. 40 DAFB. (C) Heatmap of 49 DEMs obtained from intersection of 10 vs. 25 DAFB, 25 vs. 33 DAFB, and 33 vs. 40 DAFB.

To identify the significant changes of metabolites at different development stages and during the whole growth period of sweet cherry, multivariate analysis was performed. First, volcano plots show all differential metabolites for 10 vs. 25 DAFB, 25 vs. 33 DAFB, and 33 vs. 40 DAFB, which were 167 DEMs (115 upregulated, 52 downregulated), 115 DEMs (84 upregulated, 31 downregulated), and 135 DEMs (41 upregulated, 94 downregulated), respectively (Fig 3A). Most of the DEMs accumulated from the green fruit stage (10 DAFB) to the degreening stage (25 DAFB), and from the degreening stage to the full red fruit stage (33 DAFB). However, from the full red fruit stage to the dark red stage (40 DAFB), there were many more downregulated than upregulated metabolites (41 upregulated and 94 downregulated).

The common differential metabolites among the three combinations were displayed in a Venn diagram, and a total of 49 metabolites were eligible (Fig 3B). Profiles of the 49 common metabolites during the four fruit-development stages can be observed in Fig 3C and are listed in Table 1. It shows that the majority accumulated from the green fruit to the full red fruit stage (33 DAFB) and reached the peak at 33 DAFB (Fig 3C). The metabolites were significantly involved in amino acids (12), Organic acids (6), and phenylpropanoids (5). Of these amino acids, N6, N6, N6-Trimethyl-L-lysine (Fig 3C, No.17), D-(-)-glutamine (Fig 3C, No.18), L-(-)-serine (Fig 3C, No.19), and Asp-Asp (Fig 3C, No.20) mainly show an increase trend during cherry development. L-glutamic acid (Fig 3C, No.10) peaked at maturation stage (40 DAFB). While D-(+)-Proline (Fig 3C, No.43) shows a consistent downward trend (Fig 3C and Table 1).

**Table 1:** The profile of the 49 common metabolites.

**No.**	**Name**	**Formula**	**Class**	**Molecular Weight**	**RT [min]**	**10 DAFBa**	**10 DAFBb**	**10 DAFBc**	**25 DAFBa**	**25 DAFBb**	**25 DAFBc**	**33 DAFBa**	**33 DAFBb**	**33 DAFBc**	**40 DAFBa**	**40 DAFBb**	**40 DAFBc**
1	unknown		other	103.00	0.58	1.29E+07	1.35E+07	1.61E+07	2.67E+07	2.76E+07	2.21E+07	3.19E+07	3.41E+07	3.12E+07	2.11E+07	1.67E+07	1.74E+07
2	Diethylene glycol	C_4_H_10_O_3_	other	106.06	5.89	5.35E+06	5.82E+06	5.92E+06	9.52E+06	8.74E+06	9.68E+06	1.24E+07	1.20E+07	1.23E+07	7.98E+06	7.42E+06	7.17E+06
3	N-Acetylimidazole	C_5_H_6_N_2_O	Organoheterocyclic compounds	110.05	0.92	4.55E+06	4.73E+06	3.30E+06	1.14E+07	7.14E+06	8.73E+06	1.70E+07	1.70E+07	1.73E+07	6.23E+06	3.20E+06	4.47E+06
4	1-Piperideine	C_5_H_9_N	Pyridines	83.07	2.16	2.73E+06	3.09E+06	2.63E+06	5.11E+06	5.24E+06	4.65E+06	7.10E+06	7.25E+06	5.84E+06	3.44E+06	2.94E+06	2.76E+06
5	unknown	C_8_H_4_O_3_	other	148.02	10.92	4.13E+07	4.32E+07	4.20E+07	7.50E+07	7.48E+07	6.45E+07	9.76E+07	9.37E+07	9.10E+07	5.64E+07	5.10E+07	4.79E+07
6	Monobutyl phthalate	C_12_H_14_O_4_	Organic acids	222.09	10.91	3.33E+06	3.57E+06	3.24E+06	5.99E+06	6.13E+06	5.33E+06	8.12E+06	7.84E+06	7.57E+06	4.26E+06	4.27E+06	3.85E+06
7	1,3,7-Octanetriol	C_8_H_18_O_3_	Lipids	162.13	5.89	2.35E+07	2.37E+07	2.35E+07	4.18E+07	4.06E+07	3.76E+07	5.47E+07	6.25E+07	5.51E+07	3.03E+07	2.66E+07	2.79E+07
8	D-(+)-Pantothenic acid	C_9_H_17_NO_5_	Organic acids	219.11015	4.25	7.16E+06	6.90E+06	7.73E+06	2.19E+07	2.29E+07	1.56E+07	3.17E+07	3.29E+07	2.85E+07	1.15E+07	8.05E+06	9.39E+06
9	Militarinone A	C_26_H_37_NO_6_	Lipids	459.26061	8.86	5.89E+06	6.30E+06	6.19E+06	1.19E+07	1.06E+07	1.05E+07	2.53E+07	2.33E+07	1.77E+07	8.44E+06	7.39E+06	6.76E+06
10	L-Glutamic acid	C_5_H_9_NO_4_	Amino acids	147.05275	0.84	9.36E+07	9.98E+07	9.76E+07	2.18E+08	1.78E+08	1.41E+08	3.04E+08	3.09E+08	2.96E+08	1.14E+08	9.59E+07	1.02E+08
11	unknown		other	101.96312	0.58	8.48E+06	8.46E+06	8.59E+06	1.33E+07	1.33E+07	1.22E+07	1.81E+07	1.88E+07	1.73E+07	9.55E+06	8.44E+06	7.75E+06
12	5,6-dihydroxythymidine	C_10_H_16_N_2_O_7_	Carbohydrates	276.09486	0.84	5.31E+07	5.52E+07	5.14E+07	9.68E+07	9.63E+07	8.64E+07	1.70E+08	1.62E+08	1.60E+08	6.67E+07	5.65E+07	5.46E+07
13	1-(beta-D-ribofuranosyl) thymine	C_10_H_14_N_2_O_6_	Nucleosides	258.08439	0.84	2.83E+07	2.93E+07	2.78E+07	5.15E+07	5.22E+07	4.64E+07	8.58E+07	8.62E+07	8.43E+07	3.44E+07	3.10E+07	2.80E+07
14	Dihydrothymine	C_5_H_8_N_2_O_2_	Organoheterocyclic compounds	128.05845	0.79	1.68E+06	2.83E+06	2.30E+06	8.46E+06	8.64E+06	7.85E+06	1.18E+07	1.26E+07	1.20E+07	2.21E+06	1.38E+06	5.45E+05
15	Dopachrome	C_9_H_7_NO_4_	Amino acids	193.03697	0.85	7.91E+06	7.82E+06	8.77E+06	1.35E+07	1.46E+07	1.12E+07	2.78E+07	2.80E+07	2.65E+07	6.86E+06	6.74E+06	6.22E+06
16	Dihydrouridine	C_9_H_14_N_2_O_6_	Nucleosides	246.08449	0.85	1.06E+07	9.80E+06	1.02E+07	1.56E+07	1.50E+07	1.32E+07	4.19E+07	4.22E+07	3.70E+07	4.88E+06	4.42E+06	4.23E+06
17	N6,N6,N6-Trimethyl-L-lysine	C_9_H_20_N_2_O_2_	Amino acids	188.15199	0.70	1.83E+06	1.73E+06	2.00E+06	5.00E+06	5.84E+06	5.23E+06	1.09E+07	1.12E+07	1.08E+07	1.74E+07	1.72E+07	1.78E+07
18	D-(-)-Glutamine	C_5_H_10_N_2_O_3_	Amino acids	146.06884	0.81	2.47E+07	2.86E+07	2.74E+07	3.50E+07	3.67E+07	3.19E+07	7.48E+07	7.35E+07	7.19E+07	6.82E+07	5.97E+07	5.79E+07
19	L-(-)-Serine	C_3_ H_7_ NO_3_	Amino acids	105.04232	0.82	2.06E+07	2.20E+07	2.16E+07	2.84E+07	2.99E+07	2.46E+07	5.42E+07	5.15E+07	5.23E+07	4.53E+07	3.88E+07	3.75E+07
20	Asp-Asp	C_8_H_12_N_2_O_7_	Amino acids	248.0638	1.16	5.13E+06	5.62E+06	5.33E+06	4.53E+07	4.83E+07	3.65E+07	5.82E+07	5.94E+07	5.73E+07	3.69E+07	3.38E+07	3.51E+07
21	Ethyl paraben	C_9_H_10_O_3_	Organic acids	134.03649	4.68	1.43E+06	1.51E+06	1.30E+06	5.52E+06	5.18E+06	5.68E+06	6.98E+06	7.46E+06	6.87E+06	3.70E+06	3.83E+06	3.36E+06
22	Caffeic acid	C_9_H_8_O_4_	Phenylpropanoids	180.04184	4.68	1.70E+07	1.56E+07	1.42E+07	7.03E+07	6.75E+07	6.85E+07	9.40E+07	9.15E+07	8.93E+07	4.98E+07	4.61E+07	3.92E+07
23	4-Hydroxy-2H-chromen-2-one	C_9_H_6_O_3_	Phenylpropanoids	162.03128	4.68	1.61E+07	1.46E+07	1.33E+07	6.62E+07	6.36E+07	6.47E+07	8.92E+07	8.63E+07	8.39E+07	4.69E+07	4.38E+07	3.70E+07
24	2(5H)-Furanone	C_4_H_4_O_2_	Organoheterocyclic compounds	84.02092	0.91	1.11E+07	5.22E+06	9.02E+06	1.74E+07	1.66E+07	1.46E+07	3.62E+07	3.09E+07	3.70E+07	1.79E+07	1.62E+07	1.80E+07
25	Piracetam	C_6_H_10_N_2_O_2_	Amino acids	142.0739	0.89	1.40E+07	8.73E+06	1.25E+07	2.85E+07	2.61E+07	2.57E+07	6.06E+07	5.54E+07	5.94E+07	2.42E+07	2.19E+07	2.09E+07
26	4-Amino-1,3-benzenediol	C_6_H_7_NO_2_	Phenylpropanoids	125.04754	0.94	1.29E+07	7.86E+06	1.13E+07	2.32E+07	2.00E+07	2.06E+07	4.82E+07	4.13E+07	4.79E+07	2.07E+07	1.87E+07	1.49E+07
27	L-Pyroglutamic acid	C_5_H_7_NO_3_	Amino acids	129.0423	0.83	5.09E+07	5.49E+07	5.25E+07	8.01E+07	8.27E+07	7.26E+07	1.54E+08	1.51E+08	1.49E+08	9.75E+07	8.58E+07	8.14E+07
28	N-Methyl-N-nitrosoethenamine	C_3_H_6_N_2_O	Amines	86.04831	0.80	1.52E+07	1.39E+07	1.43E+07	2.38E+07	2.35E+07	2.21E+07	4.91E+07	4.41E+07	3.56E+07	2.14E+07	1.90E+07	1.96E+07
29	Bis (4-ethylbenzylidene) sorbitol	C_24_H_30_O_6_	Carbohydrates	414.20287	8.85	1.03E+07	1.05E+07	1.00E+07	2.11E+07	2.17E+07	1.89E+07	6.10E+07	5.41E+07	4.13E+07	1.70E+07	1.54E+07	1.51E+07
30	Maleamic acid	C_4_H_5_NO_3_	Lipids	115.02669	0.80	3.05E+08	3.32E+08	3.17E+08	5.14E+08	5.20E+08	4.80E+08	9.01E+08	8.93E+08	8.03E+08	4.56E+08	4.31E+08	4.25E+08
31	(1E)-N-Hydroxy-4-(methylsulfanyl)-1-butanimine	C_5_H_11_NOS	Amines	133.05644	0.79	1.35E+07	1.41E+07	1.27E+07	2.28E+07	2.25E+07	1.98E+07	3.79E+07	3.60E+07	3.28E+07	2.12E+07	1.84E+07	1.82E+07
32	3-Methylcyclohexanethiol	C_7_H_14_S	other	130.08212	0.95	6.42E+06	7.40E+06	7.08E+06	1.27E+07	1.35E+07	1.14E+07	9.89E+06	9.25E+06	7.72E+06	5.49E+06	4.34E+06	4.92E+06
33	Sorbic acid	C_6_H_8_O_2_	Lipids	112.05221	0.95	1.05E+08	1.13E+08	9.45E+07	2.06E+08	2.17E+08	1.80E+08	1.43E+08	1.26E+08	1.33E+08	1.15E+08	7.25E+07	8.24E+07
34	Pipecolic acid	C_6_H_11_NO_2_	Organic acids	129.07878	0.95	1.05E+08	1.13E+08	9.41E+07	2.05E+08	2.16E+08	1.80E+08	1.43E+08	1.26E+08	1.33E+08	1.15E+08	7.22E+07	8.19E+07
35	N-(2-Methoxybenzoyl) glycine	C_10_H_11_NO_4_	Organic acids	209.06833	2.01	3.44E+07	3.53E+07	3.37E+07	7.12E+07	6.88E+07	5.83E+07	5.38E+07	5.38E+07	5.23E+07	1.65E+07	1.45E+07	1.42E+07
36	L-(-)-Threonine	C_4_H_9_NO_3_	Amino acids	119.0582	0.82	1.15E+07	1.17E+07	1.05E+07	2.38E+07	2.33E+07	2.00E+07	1.68E+07	1.70E+07	1.69E+07	6.58E+06	5.31E+06	5.58E+06
37	Metirosine	C_10_H_13_NO_3_	Phenylpropanoids	195.08916	1.94	5.13E+07	4.98E+07	5.16E+07	1.04E+08	1.03E+08	8.94E+07	8.21E+07	8.12E+07	8.01E+07	3.37E+07	3.17E+07	3.27E+07
38	Skatole	C_9_H_9_N	Alkaloids	131.0733	1.94	3.57E+07	3.59E+07	3.57E+07	5.97E+07	6.46E+07	5.82E+07	5.53E+07	5.42E+07	4.96E+07	2.08E+07	1.98E+07	2.28E+07
39	Ursolic acid	C_30_H_48_O_3_	Terpenoids	456.35896	13.53	7.47E+06	7.95E+06	7.56E+06	2.07E+07	2.17E+07	1.90E+07	9.10E+06	8.97E+06	7.94E+06	6.55E+05	5.35E+05	5.73E+05
40	2-phytyl-1,4-dihydroxynaphthalene	C_30_H_46_O_2_	Terpenoids	438.34835	13.53	5.81E+06	6.20E+06	5.90E+06	1.62E+07	1.75E+07	1.50E+07	7.14E+06	7.06E+06	6.15E+06	5.42E+05	4.13E+05	4.53E+05
**No.**	**Name**	**Formula**	**Class**	**Molecular Weight**	**RT [min]**	**10 DAFBa**	**10 DAFBb**	**10 DAFBc**	**25 DAFBa**	**25 DAFBb**	**25 DAFBc**	**33 DAFBa**	**33 DAFBb**	**33 DAFBc**	**40 DAFBa**	**40 DAFBb**	**40 DAFBc**
41	4-Coumaric acid	C_9_H_8_O_3_	Phenylpropanoids	164.04701	4.94	4.20E+07	4.14E+07	4.05E+07	6.84E+07	7.17E+07	6.00E+07	5.96E+07	5.06E+07	4.83E+07	2.25E+07	1.92E+07	1.90E+07
42	4-Methyl-2-oxo-2H-chromen-7-yl β-D-glucopyranoside	C_16_H_18_O_8_	Amino acids	338.0991	4.93	1.49E+07	1.51E+07	1.46E+07	2.37E+07	2.27E+07	2.09E+07	1.80E+07	1.80E+07	1.74E+07	7.56E+06	6.44E+06	6.48E+06
43	D-(+)-Proline	C_5_H_9_NO_2_	Amino acids	115.06328	0.87	2.71E+07	2.42E+07	2.41E+07	1.39E+07	1.26E+07	1.22E+07	9.03E+06	8.91E+06	7.97E+06	4.42E+06	3.94E+06	3.65E+06
44	unknown	C_13_H_17_N_3_O_10_	other	375.09021	1.30	1.58E+07	1.67E+07	1.62E+07	1.29E+07	1.45E+07	1.01E+07	1.66E+07	2.01E+07	1.64E+07	2.25E+06	2.03E+06	2.03E+06
45	Piperidine	C_5_H_11_N	Alkaloids	85.08948	1.23	1.97E+07	1.97E+07	2.06E+07	2.95E+07	2.56E+07	2.44E+07	4.55E+07	4.10E+07	3.60E+07	2.48E+06	2.25E+06	2.56E+06
46	L-(+)-Valine	C_5_H_11_NO_2_	Amino acids	117.07893	0.92	5.80E+07	5.46E+07	5.57E+07	6.45E+07	6.43E+07	5.95E+07	9.72E+07	8.95E+07	8.35E+07	6.73E+06	5.54E+06	5.11E+06
47	(2S)-4-Methyl-2-({[(3S,4S,5R)-2,3,4-trihydroxy-5-(hydroxymethyl) tetrahydro-2-furanyl]methyl}amino) pentanoic acid	C_12_H_23_NO_7_	Organic acids	293.14654	1.68	2.69E+07	2.62E+07	2.53E+07	3.48E+07	3.24E+07	2.82E+07	4.05E+07	3.95E+07	3.71E+07	2.55E+06	2.15E+06	1.99E+06
48	Dulcitol	C_6_H_14_O_6_	Carbohydrates	182.08084	0.87	1.83E+08	1.86E+08	2.11E+08	1.39E+07	1.48E+07	1.44E+07	8.15E+05	7.08E+05	7.19E+06	2.66E+08	4.29E+08	4.53E+08
49	N-Acetylneuraminic acid	C_11_ H_19_NO_9_	Carbohydrates	309.10494	0.89	2.48E+07	2.50E+07	2.29E+07	1.52E+07	1.43E+07	1.31E+07	2.32E+07	2.30E+07	2.35E+07	1.92E+07	1.87E+07	1.99E+07

RT: Retention time. DAFB: Days after full bloom. Differentially expressed metabolites (DEMs) were identified using thresholds of VIP > 1 and *p* < 0.05.

**Fig 4:**
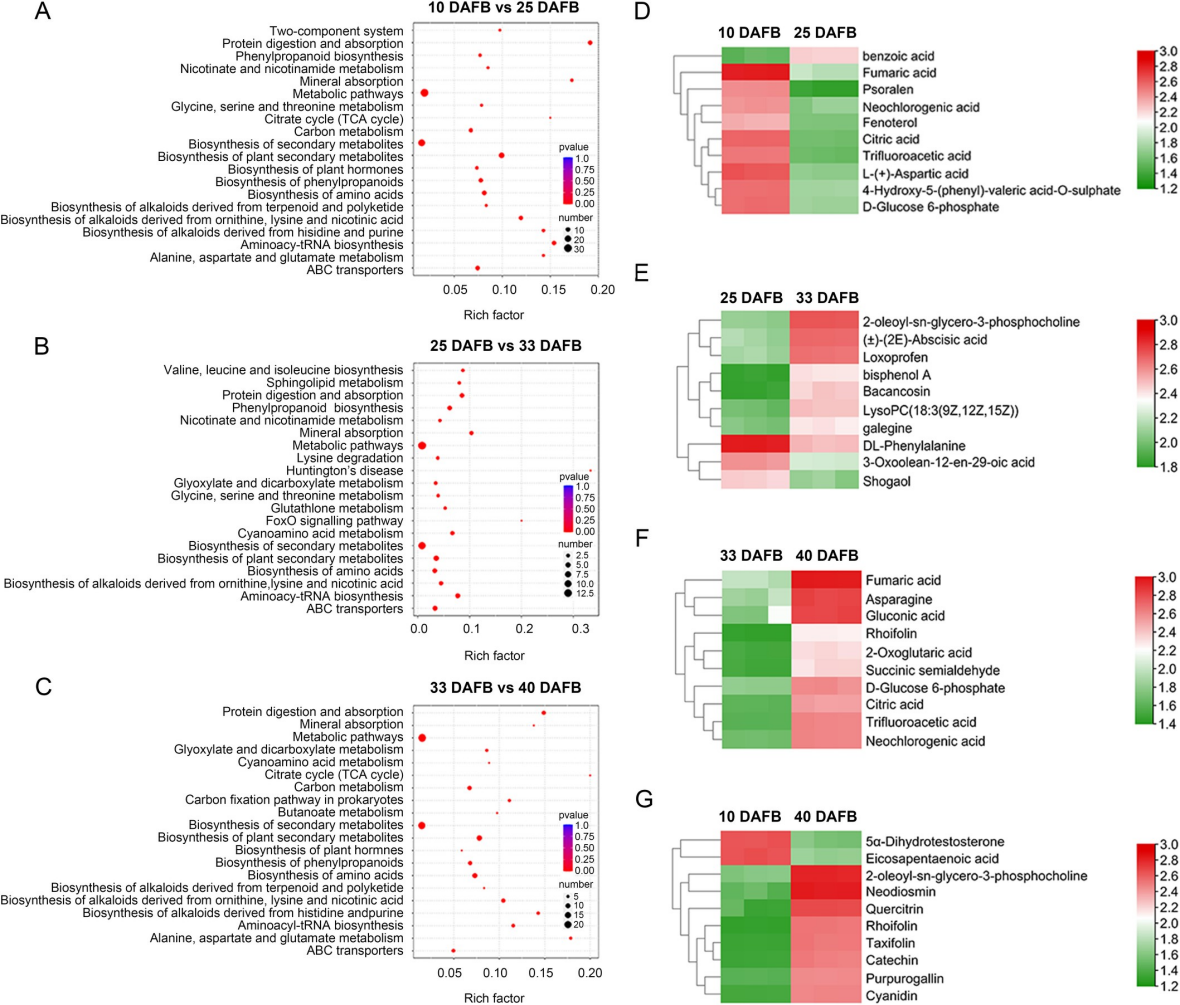
KEGG pathway enrichment analysis of DEMs in (A) 10 vs. 25 DAFB, (B) 25 vs. 33 DAFB, and (C) 33 vs. 40 DAFB. Heatmaps of top 10 DEMs in (D) 10 vs. 25 DAFB, (E) 25 vs. 33 DAFB, (F) 33 vs. 40 DAFB, and (G) 10 vs. 40 DAFB.

Furthermore, KEGG pathway analysis of DEMs was performed to identify significantly enriched metabolic pathways. Interestingly, although there were distinct differences in the top 20 enriched pathways across the three combinations (10 vs. 25 DAFB, 25 vs. 33 DAFB, and 33 vs. 40 DAFB), the most significantly enriched pathways were involved in metabolic pathways and biosynthesis of secondary metabolites (Fig 4A–4C). To further investigate the metabolites that changed the most during sweet cherry development, heatmaps were drawn for the 10 most noticeable metabolites with significant differences in the three combinations (Fig 4D–4F and S4 File). The top 10 DEMs in the 10 vs. 25 DAFB combination included five organic acids (citric acid, trifluoroacetic acid, fumaric acid, and neochlorogenic acid, benzoic acid), one amine (fenoterol), one carbohydrate (D-glucose 6-phosphate (G6P)), one furan (psoralen), and one amino acid (L-(+)-aspartic acid), and one other (4-hydroxy-5-(phenyl)-valeric acid-O-sulphate) (Fig 4D). Interestingly, except for benzoic acid, the expression of 9 other metabolites in 10 DAFB stage was significantly higher than that in 25 DAFB stage (Fig 4D). In the 25 vs. 33 DAFB combination, the top 10 DEMs consisted of two terpenoids (3-oxoolean-12-en-29-oic acid, (±) -(2E)-abscisic acid), two lipids (2-oleoyl-sn-glycero-3-phosphocholine, lysoPC (18:3(9Z,12Z,15Z))), two phenylpropanoids (shogaol, bisphenol A), one carbohydrate (bacancosin), one organic acid (loxoprofen), one alkaloid (galegine), and one amino acid (DL-phenylalanine) (Fig 4E). Here, shogaol, 3-oxoolean-12-en-29-oic acid and DL-phenylalanine highly expressed in 25 DAFB instead of 33 DAFB, which is contrary to the trend of other metabolites expression (Fig 4E). For the 33 vs. 40 DAFB combination, the top 10 DEMs contained five organic acids (trifluoroacetic acid, fumaric acid, neochlorogenic acid, citric acid, 2-oxoglutaric acid), two carbohydrates (D-glucose 6-phosphate, gluconic acid), one amino acid (asparagine), one lipid (succinic semialdehyde), and one flavonoid (rhoifolin) (Fig 4F). The expression of all these metabolites in 40 DAFB stage was significantly higher than that in 33 DAFB stage (Fig 4F). We also focused on the DEMs in the 10 vs. 40 DAFB combination to view the changes of metabolites during the whole growth period (Fig 4G). For this combination, the top 10 DEMs were divided into three categories: six flavonoids (neodiosmin, quercitrin, rhoifolin, taxifolin, catechin, cyanidin), three lipids (eicosapentaenoic acid, 5α-dihydrotestosterone, 2-oleoyl-sn-glycero-3-phosphocholine), and one phenylpropanoids (purpurogallin) (Fig 4G). Compared with 10 DAFB stage, all the six flavonoids showed significantly upregulation trend in 40 DAFB stage (Fig 4G).

### Changes in transcriptome profile and association analysis of DEMs and DEGs

**Fig 5:**
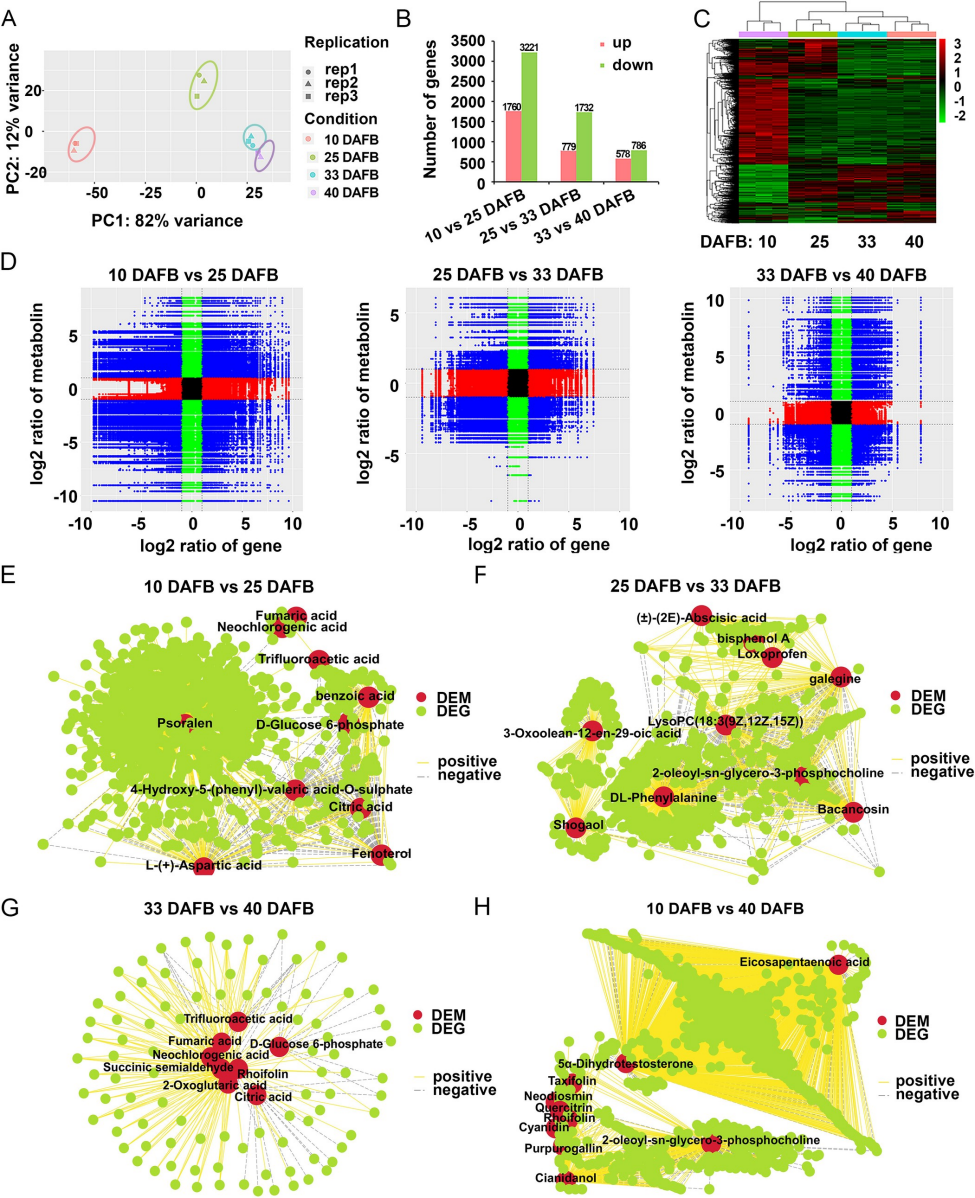
Correlation analysis of metabolomic and transcriptomic data. (A) PCA plots of differentially expressed genes (DEGs) identified from different developmental stages of sweet cherry. (B) Number of DEGs among different fruit-development stages. (C) Heatmap of all identified DEGs at four stages. Red and green indicate increased and decreased gene transcript levels, respectively. (D) Nine-quadrant diagrams show the correlation of compounds (obtained from metabolomic analysis) and genes (identified from RNA-seq) in sweet cherry. Blue, green, red, and black points indicate DEGs and DEMs pairs, non-DEGs and DEM pairs, DEGs and non-DEM pairs, and non-DEGs and non-DEM pairs, respectively. (E-H) Association analysis of transcriptomic and metabolomic variation. Connection network between top 10 DEMs and screened DEGs (|log2FoldChange| > 1, *p* < 0.05, and FPKM > 10) in (E) 10 vs. 25 DAFB, (F) 25 vs. 33 DAFB, (G) 33 vs. 40 DAFB, and (H) 10 vs. 40 DAFB. Red and green ovals in nodes represent DEMs and DEGs, respectively. Edges represent “relationships” between any DEMs and DEGs, yellow and gray represent positive and negative correlations, respectively, as determined by absolute value of |r| > 0.90.

RNA-seq analysis of sweet cherry samples during different developmental stages was conducted to quantify gene expression changes. PCA clearly separated the 10 and 25 DAFB from 33 and 40 DAFB samples based on PC1, with PC1 contributing 82% variation, making it the dominant component. While, 33 and 40 DAFB samples were located close to each other in both PC1 and PC2 (Fig 5A). A total of 6724 significant DEGs were identified among the three compared combinations. Specifically, there were 4981, 2511, and 1364 DEGs were identified for 10 vs. 25 DAFB, 25 vs. 33 DAFB, and 33 vs. 40 DAFB comparison groups (Fig 5B). The number of DEGs was much less in 33 vs. 40 DAFB than in the other two combinations. The expression levels of the 6724 DEGs are shown in a heatmap in Fig 5C, and they exhibit different expression patterns. The variations of all DEMs and DEGs in each comparison are illustrated by nine-quadrant diagrams (Fig 5D), with |r| > 0.80. The DEMs and DEGs shown in quadrants 3 and 7 are positively associated, while those in quadrants 1 and 9 are negatively associated (Fig 5D). Compared with 10 vs. 25 DAFB, the number of DEMs and DEGs in 25 vs. 33 DAFB and 33 vs. 40 DAFB largely decreased, indicating strong changes from green fruit to the degreening stage. To investigate the metabolites that changed the most, we used correlation network diagrams (|r| > 0.90) to further investigate the connection between the top 10 altered DEMs and major DEGs (|log_2_FoldChange| > 1, *p* < 0.05, and FPKM > 10) in 10 vs. 25 DAFB (Fig 5E and S5 File), 25 vs. 33 DAFB (Fig 5F and S5 File), 33 vs. 40 DAFB (Fig 5G and S5 File), and 10 vs. 40 DAFB (Fig 5H and S5 File). The correlation analysis suggests that these DEGs might play a direct or indirect regulatory role in key DEM metabolism over the whole developmental period of sweet cherry.

### Expression patterns associated with sugar, organic acid, and flavonoid metabolism

**Fig 6:**
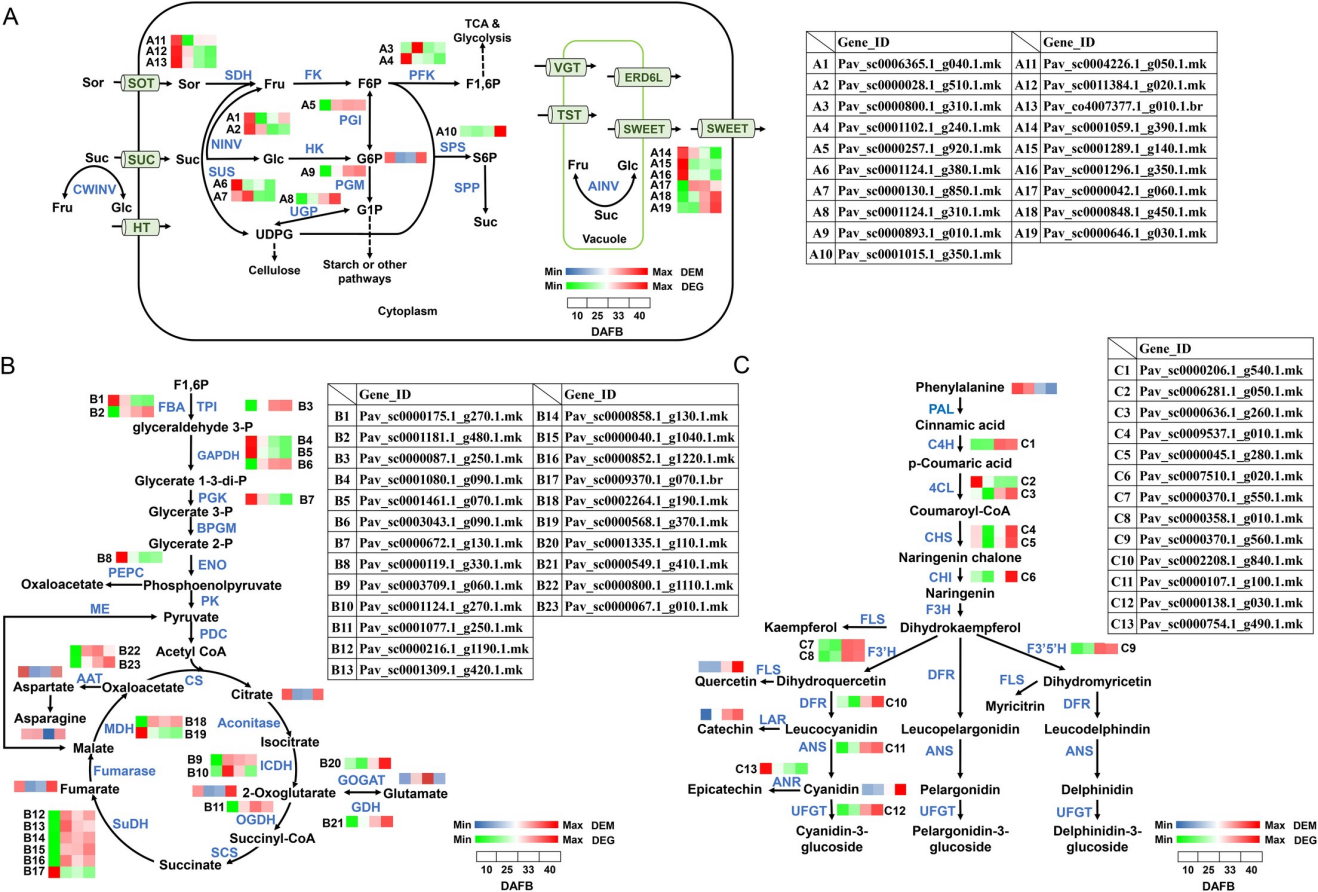
Expression profiles of DEGs and DEMs associated with (A) sugar metabolism, (B) organic acid metabolism, and (C) flavonoid metabolism.

To further explore transcriptional regulations in sweet cherry development and quality formation, major DEGs that were highly correlated (|r| > 0.90) with the top 10 DEMs in each of the four comparisons (10 vs. 25 DAFB, 25 vs. 33 DAFB, 33 vs. 40 DAFB, 10 vs. 40 DAFB) were assigned to metabolic pathways (Fig 6). According to the taxonomic properties of the top 10 DEMs in each comparison, the highly related DEGs assigned to the sugar, organic acid, and flavonoid metabolism pathways were further investigated (Fig 6 and S6 File).

For sugar metabolism: Sor, sorbitol; Fru, D-fructose; Suc, sucrose; Glc, D-glucose; F6P, fructose-6-phosphate; G6P, glucose-6-phosphate; F1,6P, Fructose 1,6-bisphosphate; S6P, sorbitol-6-phosphate; G1P, glucose-1-phosphate; UDPG, UDP-D-glucose; PFK, phosphofructokinase; NINV, neutral invertase; SUS, sucrose synthase; PGI, phosphoglucoisomerase; PGM, phosphoglucomutase; UGP, UDPG-pyrophosphorylase; SPS, sucrose-phosphate synthase; SOT, sorbitol transporter; SUC, sucrose carrier or transporter; HT, hexose transporter; VGT, vacuole glucose transporter; TST, tonoplast sugar transporter; ERD6L, early response to dehydration-6-like; SWEET, sugars will eventually be exported transporter.

For organic acids metabolism: FBA, fructose-bisphosphate aldolase; TPI, triose-phosphate isomerase; GAPDH, glyceraldehyde-3-phosphate dehydrogenase; PGK, phosphoglycerate kinase; PEPC, phosphoenolpyruvate carboxylase; ICDH, isocitrate dehydrogenase; GOGAT, glutamate synthase; GDH, glutamate dehydrogenase; OGDH, 2-oxoglutarate dehydrogenase; SuDH, succinate dehydrogenase; AAT, aspartate aminotransferase; MDH, malate dehydrogenase.

For flavonoid metabolism: C4H, cinnamate 4-monooxygenase; 4CL, 4-coumarate-CoA ligase; CHS, chalcone synthase; CHI, chalcone isomerase; F3’H, flavonoid 3’-monooxygenase; F3’5’H, flavonoid 3’,5’-hydroxylase; DFR, dihydroflavonol 4-reductase; ANS, anthocyanidin synthase; ANR, anthocyanidin reductase; UFGT, UDP-glucose: flavonoid 3-*O*-glucosyltransferase.

For sugar metabolism, the major metabolite G6P was found significantly downregulated in the 10 vs. 25 DAFB comparison, but significantly accumulated from 33 DAFB to 40 DAFB stage (Fig 6A). The content of G6P in 40 DAFB stage was significantly higher than that in 10 DAFB stage (Fig 6A and S6 File). Enzymes closely involved in G6P formation and metabolism include hexokinase (HK), phosphoglucoisomerase (PGI), and phosphoglucomutase (PGM). The transcript level of *PGI* (A5) obviously increased from 10 to 25 DAFB, and then maintained at higher levels at further developmental stages. The expression of sucrose-phosphate synthase (*SPS*, A10) showed the total opposite mode from *PGI* (A5), staying at a low level up to 33 DAFB and then increasing sharply from full red to dark red stage. The expression of UDPG-pyrophosphorylase (*UGP*, A8), *PGM* (A9), and Sugar Will Eventually be Exported Transporter (*SWEETs*, A18, A19) increased during fruit development and ripening. One neutral invertase (*NINV*, A1) and one sorbitol transporter (*SOT*, A11) exhibited an initial decreasing trend and increased in the late stage (Fig 6A). The expression patterns of one *NINV* (A2), one phosphofructokinase (*PFK*, A4), one sucrose synthase (*SUS*, A6), two SOT (A12, A13), and three *SWEETs* (A14, A15, A16) were highest in the early green stage, but maintained low levels during fruit ripening (Fig 6A).

For organic acid metabolism, some major metabolites were enriched in this pathway, including citrate, 2-oxoglutarate, glutamate, fumarate, aspartate, and asparagine (Fig 6B and S6 File). The content of citrate, 2-oxoglutarate, fumarate, and aspartate obviously decreased from the green fruit stage (10 DAFB) to the degreening phase (25 DAFB), and increased from full red (33 DAFB) to dark red (40 DAFB). On the contrary, the glutamate content notably accumulated from 10 to 33 DAFB, but decreased sharply during fruit ripening. At the transcript level, one fructose-bisphosphate aldolase (*FBA*, B1), two glyceraldehyde-3-phosphate dehydrogenases (*GAPDHs*, B4 and B5), and phosphoglycerate kinase (*PGK*, B7) decreased with fruit development, while another *FBA* (B2), glutamate dehydrogenase (*GDH*, B21), and one aspartate aminotransferase (*AAT*, B23) increased and peaked in the latest stage. Meanwhile, the expression of phosphoenolpyruvate carboxylase (*PEPC*, B8), one succinate dehydrogenase (*SuDH*, B17), and one malate dehydrogenase (*MDHs*, B19) significantly dropped from the green fruit to the degreening phase, and remained at those low levels during development. In contrast, levels of one *GAPDH* (B6), one isocitrate dehydrogenase (*ICDHs*, B9), 2-oxoglutarate dehydrogenase (*OGDH*, B11), five *SuDHs* (B12-B16), and one *AAT* (B22) significantly increased from the green fruit to degreening stage, and then maintained those higher levels till fruit ripening. However, the level of *ICDH* (B10) was highest in 25 DAFB fruit, whose expression mode was distinguished from the others (Fig 6B).

For flavonoid metabolism, major metabolites such as quercetin, catechin, and cyanidin, were remarkably increased during fruit development, in contrast to their precursor phenylalanine (Fig 6C and S6 File). The trend of cyanidin was consistent with that of anthocyanin concentration (Fig 1E). In this study, expression of almost all selected DEGs involved in the flavonoid metabolism pathway peaked at 33 or 40 DAFB, including cinnamate-4-hydroxylase (*C4H*, C1), one of two 4-coumarate-CoA ligases (*4CL*, C3), chalcone synthase (*CHS*, C4 and C5), chalcone isomerase (*CHI*, C6), flavonoid 3’-monooxygenase (*F3’H*, C7 and C8), flavonoid 3’,5’-hydroxylase (*F3’5’H*, C9), dihydroflavonol 4-reductase (*DFR*, C10), anthocyanidin synthase (*ANS*, C11), and UDP-glucose: flavonoid 3-*O*-glucosyltransferase (*UFGT*, C12), which may contribute to the significant accumulation of flavonoid compounds and the reduction of phenylalanine. In contrast, the levels of one *4CL* (C2) and anthocyanidin reductase (*ANR*, C13) reduced during fruit development. The expression level of ANR was opposite to the trend of cyanidin content (Fig 6C).

## Discussion

Studying fruit quality not only helps us to select a breeding program by exploring the most promising fruit genotypes, but also helps to improve the fruit quality, health-promoting components, commodity status, and market value [6,33]. As a seasonal fruit, sweet cherry is highly edible and has ornamental value. The sweetness of the fruit is mainly determined by soluble sugars, and the acidity is controlled by organic acids [34]. The sugar/acid ratio determines the fruit flavor [10]. Anthocyanin accumulation is the decisive factor in the red coloration of sweet cherry [4,25]. Determining the metabolites of sweet cherry is critical to study the formation of fruit quality. Metabolomics, including targeted and untargeted metabolomics techniques, has been regarded as an effective way of identifying and quantifying metabolites in various plant tissues or organizations, such as cassava [35], loquat [36], apple [9], and strawberry [29]. In sweet cherry, some specific metabolites have mainly been studied, especially sugars, acids, and phenolic compounds [1,34]. Recently, transcriptome, proteome, and metabolome profiling were employed to detect the responses of sweet cherry fruit to exogenously applied calcium, indicating that calcium contributed to fruit ripening [37]. Here, the untargeted metabolome technique was applied to investigate metabolomic variations during the sweet cherry fruit development processes, and a total of 263 significant DEMs were identified (Fig 2 and S2 File). Moreover, significant differences existed in these metabolites between different groups (Figs 2A and S1). These data provide a wide-scale metabolomic perspective of sweet cherry fruit and help us to better understand the metabolic basis of fruit quality traits.

In this study, the green fruit, degreening, full red, and dark red stages were shown at 10, 25, 33, and 40 DAFB, respectively (Fig 1A). Obvious differences were shown between the top 10 DEMs at each stage of development (Fig 4D–4G). In the 10 vs. 25 DAFB comparison, 9 of the top 10 DEMs showed significant downregulation (Fig 4D). Seven of the top 10 DEMs were significantly upregulated in the 25 vs. 33 DAFB comparison. These major metabolites changed 10 to 33 DAFB and may be mainly responsible for the increased cell division and expansion [29]. In the 33 vs. 40 DAFB comparison, the top 10 DEMs were significantly upregulated (Fig 4F), suggesting that they are the main reason for the accumulation of nutrients during fruit ripening. From green fruit (10 DAFB) to dark red (40 DAFB), the most significant change was in the accumulation of flavonoids (6/10 top DEMs), which may contribute to the coloration as well as have high antioxidant activity [24,38]. These results reveal that differentially changed metabolites were obvious at different development stages of sweet cherry, which was probably the main reason for the quality variations at different stages.

Fruit flavor and taste are controlled by the accumulation of primary metabolites, mainly sugars and organic acids. In the present study, G6P was significantly downregulated in the 10 vs. 25 DAFB comparison, and upregulated in 33 vs. 40 DAFB (Fig 6A). At the same time, six significantly changed metabolites (citrate, 2-oxoglutarate, fumarate, glutamate, aspartate, and asparagine) were observed in the organic acid metabolism pathway (Fig 6B). In plum fruit, the conversion of fructose to G6P may be due to increased PGI activity [10]. In apple, 2-oxoglutaric acid, as a crucial intermediate of the tricarboxylic acid cycle (TCA), was closely related to sugars, several amino acids, flavonoids, and nucleotides [9]. Here, 2-oxoglutarate was also identified as a major metabolite in sweet cherry (Fig 6B), which may be quite important for fruit quality. In addition, fruit quality is also influenced by the accumulation of amino acids [35]. A previous study revealed that alanine, threonine, and serine are related to sweetness in grapes, while glutamate and aspartate contribute to sour taste [13]. Asparagine and alanine affect the taste of soybean [14]. In the present work, most amino acids, are mainly accumulated at 30 or 40 DAFB stage, including glutamate, aspartate, and asparagine (Fig 6B). These results indicate that the significant variation in amino acids in sweet cherry can potentially influence fruit quality.

The remarkable changes in sugars and organic acids of primary metabolites may be closely influenced by related DEGs. In sugar metabolism, SOT transports sorbitol into cytosol, then sorbitol dehydrogenase (SDH) catalyzes Sor to form fructose. On the other hand, Suc can be converted to Fru and glucose by NINV, or to Fru and UDP-D-glucose (UDPG) by SUS. Afterwards, the resulting Glc and Fru are phosphorylated to G6P and fructose-6-phosphate (F6P) by HK and fructokinase (FK), respectively. The interconversion between G6P and F6P is catalyzed by PGI in a reversible reaction [39–41]. Seven enzyme systems were used by Beaver et al. [42] to evaluate isozyme divergence among 36 cherry species: PGI, PGM, 6-phosphogluconate dehydrogenase (6-PGD), ICDH, shikimate dehydrogenase (SKDH), leucine aminopeptidase (LAP), and MDH. In sour cherry fruits, transport mediated by PcSOT1 and PcSOT2 affected the accumulation of sorbitol and dry matter [43]. However, coding genes for sugar and organic acids in sweet cherry remain largely uncharacterized. In this study, the transcript levels of *SOT* (A11-A13), *PFK* (A3, A4), *NINV* (A1, A2), and *SUS* (A6, A7) were high at the early stage of sweet cherry fruit development (Fig 6A), which may be responsible for the efficient utilization of imported sucrose and sorbitol for fruit growth. At that sage, sugar accumulation stayed at low levels. At the late stage, sucrose accumulation was enhanced, consistent with the elevated expression of *PGI* (A5), *UGP* (A8), *PGM* (A9), and *SPS* (A10) (Fig 6A).

Finally, the SWEET genes can also influence sugar concentration, with the function of transporting sugars across cell membranes [44]. In apple, *MdSWEET2e*, *MdSWEET9b*, and *MdSWEET15a* have been demonstrated significantly to be associated with sugar content [45]. In tonoplasts of *Arabidopsis* leaf and root cells, *AtSWEET16* can transport sucrose, Glc, and Fru [46], but *AtSWEET17* appeared to work on Fru specifically [47]. In addition, *SWEETs* were also involved in some other physiological mechanisms, including nectar secretion, phloem loading, seed nutrient filling, and pathogen nutrition [44,48]. Here, six *SWEETs* (A14-A19) were identified and showed different expression trends, which may be due to the differences in the sugars they transport. For organic acid metabolism, most related DEGs located before the TCA cycle showed the highest expression at 10 DAFB (*FBA* (B1), *GAPDH* (B4, B5), *PGK* (B7), *PEPC* (B8)), while the transcript levels of most DEGs in the TCA cycle exhibited the opposite trend (Fig 6B). PEPC plays an important role in plant cells; it catalyzes irreversible β-carboxylation of phosphoenolpyruvate to form oxaloacetate and Pi [49]. In cherry, PEPC was detected in both flesh and skin, and declined during development [50]. In apple, the combination of cytosolic malate dehydrogenase 1 (cMDH1) and NADP-dependent malic enzyme (NADP-cME) can control malate synthesis [51]. In this study, *PEPC* (B8) and one *MDH* (B19) showed a consistent tendency of downregulated expression, which can decrease the content of acids by reducing malate during sweet cherry development (Fig 6B). In addition, GDH was shown to be involved in glutamate synthesis and degradation processes during tomato fruit ripening and participate in the control of metabolic composition [52]. Here, the expression of glutamate synthase (*GOGAT*, B20) and *GDH* (B21) changed significantly at each stage and peaked at 40 DAFB, which may responsible for the organic acid metabolism and affect fruit nutrient value.

The changes of secondary metabolites were particularly significant during the development of sweet cherry, especially flavonoids such as quercetin, catechin, and cyanidin (Fig 6C). The most prevalent plant flavonoids include flavonols [53,54], flavones [55,56], flavanols [57,58], and flavanones [59]. Compared with green fruit stage (10 DAFB), the contents of phenylpropanoids, alkaloids, and terpenoids were reduced in dark red stage (40 DAFB), which was opposite to the changes of flavonoids (S3 File). A previous study evaluated 22 sweet cherry accessions with regards to their sensory, physicochemical, and bioactive compound traits, revealing that all genotypes were rich in polyphenols [24]. Increased concentrations of these flavonoids may account for fruit color formation and antioxidant activity. Phenylalanine ammonia-lyase (PAL), CHS, CHI, and flavanone 3-hydroxylase (F3H), which were identified in the early steps of the flavonoid biosynthesis pathway, help to generate common precursors [60,61], and there were two *CHS* (C4, C5), one *CHI* (C6), and *C4H* (C1) and *4CL* (C3) showing high transcript levels in the fruit ripening stage in this study. In the late biosynthesis steps, flavonol synthase (FLS) contributed to the production of flavonol derivatives such as quercetin, kaempferol, and myricetin [61]. ANR and leucoanthocyanidin reductase (LAR) were characterized as key enzymes in producing proanthocyanins such as catechin and epicatechin [18]. Previous studies on tomato and tea revealed that DFR played a key role in generating anthocyanidins and catechins [62,63]. In ‘Hong Deng’ sweet cherry, the transcript levels of *PacDFR*, *PacANS*, and *PacUFGT* were closely related to anthocyanin biosynthesis during fruit development [4,64]. *AaLDOX* (*ANS*) was identified as a pivotal gene controlling the accumulation of anthocyanin in kiwifruit, and the *ANS* (C11) in Fig 6C was the same gene reported in our previous study of sweet cherry [26,65]. In our previous results, *PacANS*, *Pac4CL2*, and 11 transcription factors (TFs) were shown to regulate anthocyanin synthesis by transcription levels in ‘Hong Deng’ sweet cherry [26]. *UFGT* participates in anthocyanin biosynthesis, acting as the decisive gene in various fruits, including apple [66], red-skinned pear [22], and grape [67]. Clustering analysis showed that the selected *DFR* (C10), *ANS* (C11), and *UFGT* (C12) here were the same as those in ‘Hong Deng’, and their expression patterns were in accordance with anthocyanin accumulation (Fig 1E). These results provide reliable evidence demonstrating the scientific validity and effectiveness of the DEG screening method.

## Conclusions

In summary, the composition and content of different metabolites lead to significant changes in sweet cherry fruit quality traits during development and ripening. Taste, color, and nutrition are the vital components of fruit quality. The major metabolites, which exhibit significant variations among the green fruit, degreening, full red, and dark red stages, may be the main reason for the different aspects of fruit quality in different phases. In addition, combined metabolome and transcriptome analysis was performed, indicating significant correlations among sugars, acids, flavonoid metabolites, and related genes. These related DEGs may play important roles in fruit growth, development, and ripening processes. These results provide a broader view and a comprehensive analysis of the metabolomic variation of the sweet cherry metabolome as well as the molecular and metabolic basis of fruit quality traits.

## Supporting information

S1 Fig The PLS-DA score plot and OPLS-DA permutation test charts in all developmental stages. (PDF)

S1 File LC–MS/MS mass chromatograms of ten representative metabolites. (PDF)

S2 File The profile of the 263 significant differentially expressed metabolites (DEMs). (XLSX)

S3 File The profile of the primary and secondary metabolites. (XLSX)

S4 File The top 10 altered DEMs in 10 vs. 25 DAFB, 25 vs. 33 DAFB, 33 vs. 40 DAFB, and 10 vs. 40 DAFB. (XLSX)

S5 File Association analysis of transcriptomic and metabolomic variation (|r| > 0.90). (XLSX)

S6 File Expression profiles of DEGs associated with sugar metabolism, organic acid metabolism, and flavonoid metabolism. (XLSX)
