# Supplementary material for: Integrative analyses of metabolome and transcriptome reveals metabolomic variations and candidate genes involved in sweet cherry (Prunus avium L.) fruit quality during development and ripening
Source: PLoS One. 2021 Nov 15;16(11):e0260004. doi: 10.1371/journal.pone.0260004 (PMC8592472; doi:10.1371/journal.pone.0260004)
Supplement: S1 Fig — (PDF) [file pone.0260004.s001.pdf]

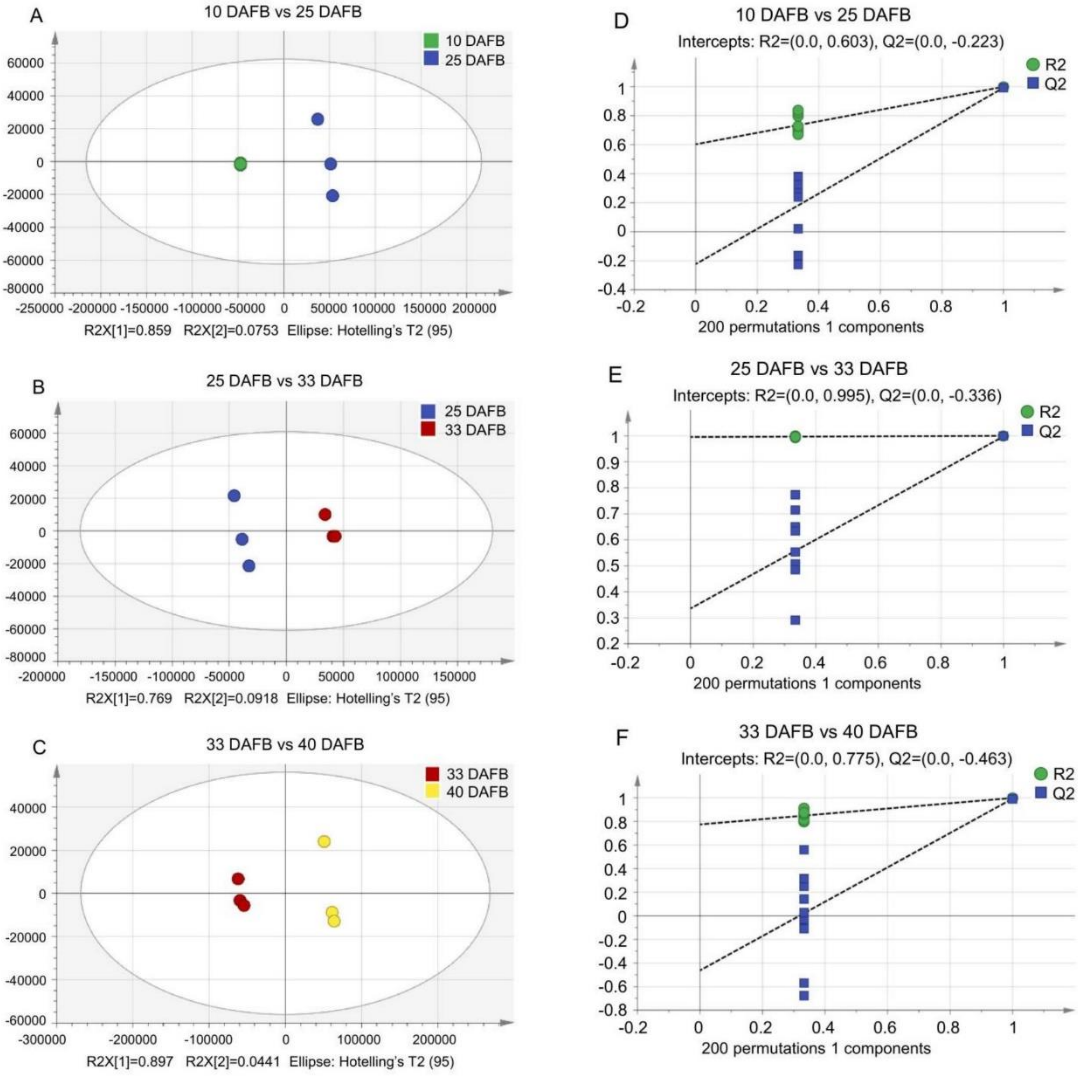

S1 Fig. The PLS-DA score plot and OPLS-DA permutation test charts in all developmental stages. PLS-DA score plot charts in (A) 10 vs. 25 DAFB, (B) 25 vs. 33 DAFB, and (C) 33 vs. 40 DAFB. The OPLS-DA permutation test charts in (D) 10 vs. 25 DAFB, (E) 25 vs. 33 DAFB, and (F) 33 vs. 40 DAFB.
