# Supplementary material for: Integrative analyses of metabolome and transcriptome reveals metabolomic variations and candidate genes involved in sweet cherry (Prunus avium L.) fruit quality during development and ripening
Source: PLoS One. 2021 Nov 15;16(11):e0260004. doi: 10.1371/journal.pone.0260004 (PMC8592472; doi:10.1371/journal.pone.0260004)
Supplement: S1 File — (PDF) [file pone.0260004.s002.pdf]

Compounds

Structure	Name	RT [min]	Formula	Molecular Weight	Group Areas
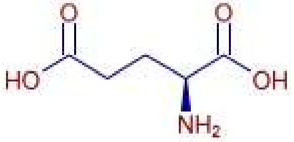	L-Glutamic acid	0.84	C5 H9 N O4	147.0528	5.62e5.99e5.86e .31e1.07e13.43e .83e1.85e1.78e15.82e5.75e5.12e

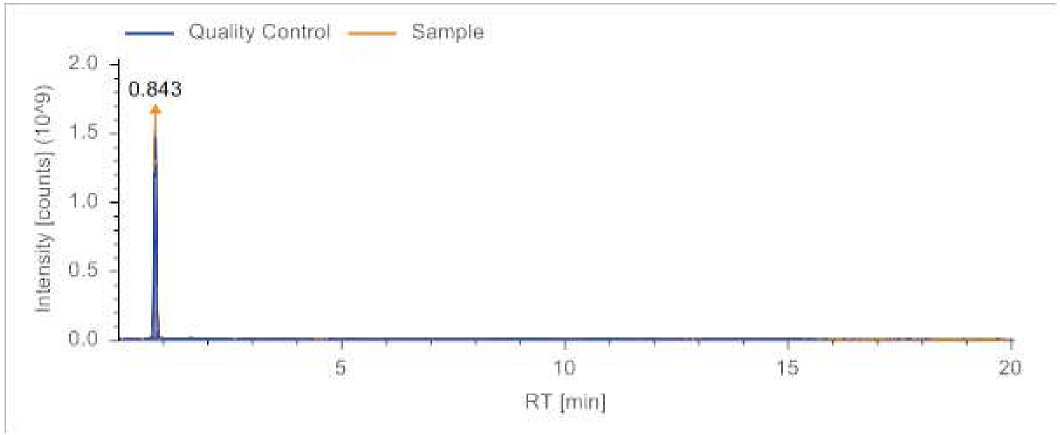

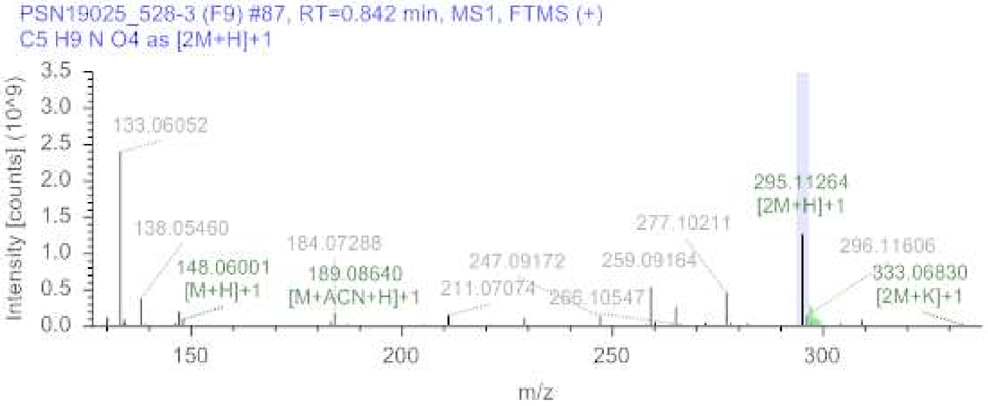

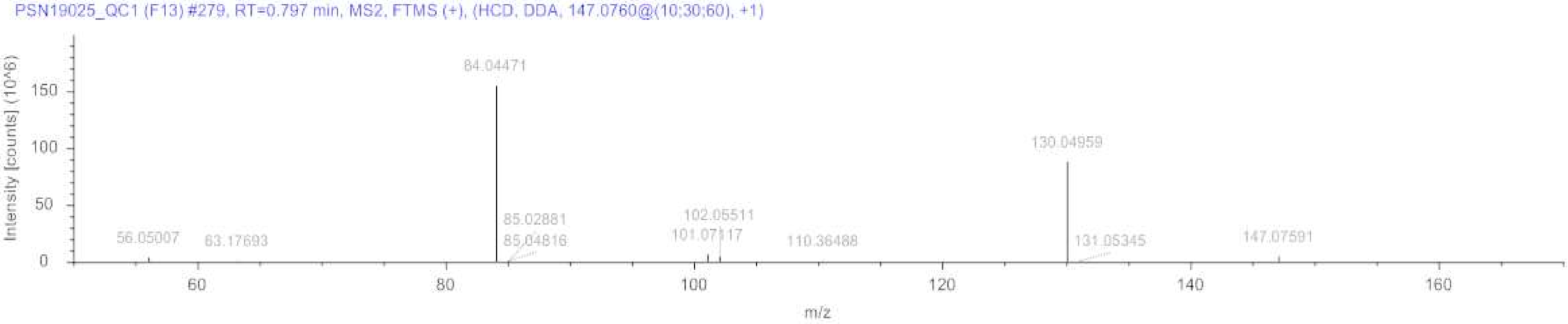

© Reported with Compound Discoverer 3.2

Structure	Name	RT [min]	Formula	Molecular Weight	Group Areas
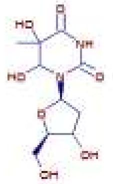	5,6-dihydroxythymidine	0.84	C10 H16 N2 O7	276.0949	3.18e93.31e93.09e95.81e95.78e95.18e9.02e13.74e9.63e94.00e93.39e93.27e

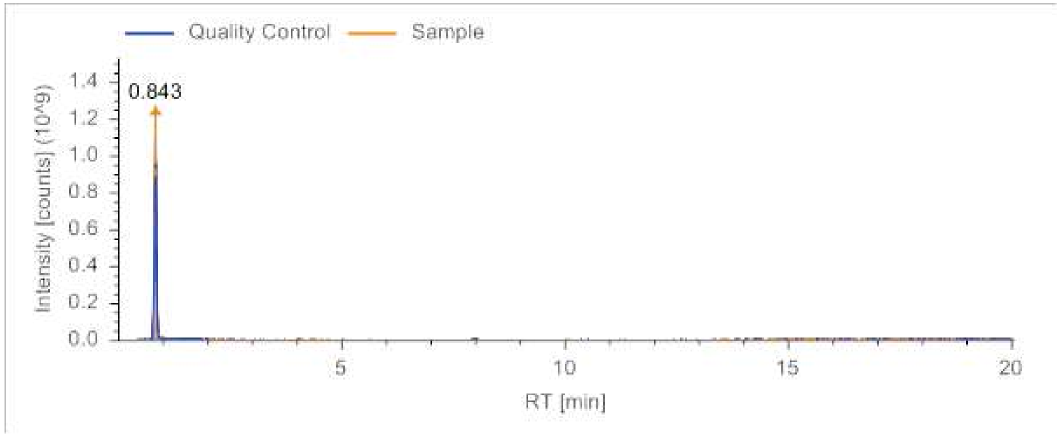

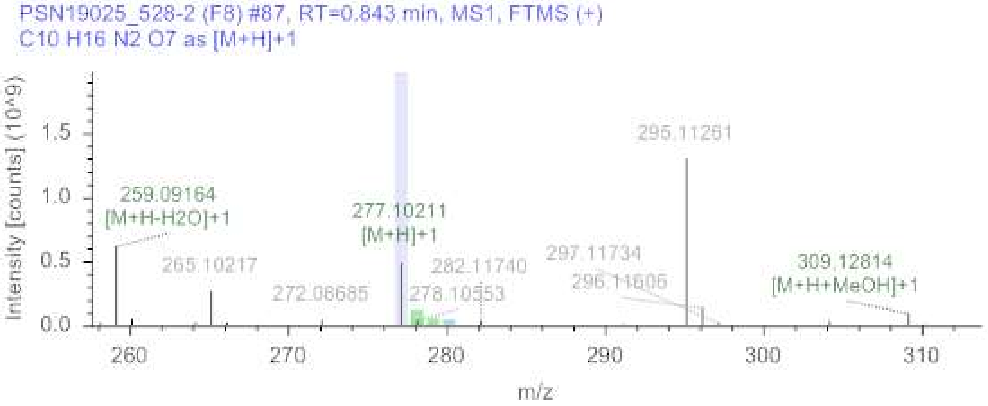

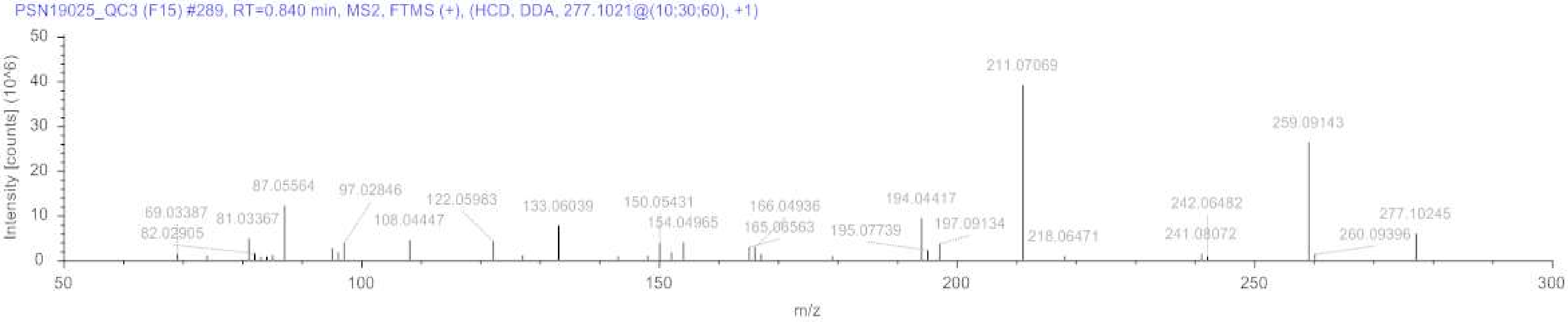

© Reported with Compound Discoverer 3.2

Compounds

Structure	Name	RT [min]	Formula	Molecular Weight	Group Areas											
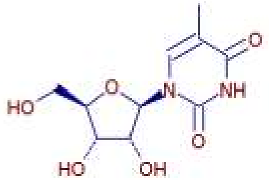	1-(beta-D-ribofuranosyl)thymine	0.84	C10 H14 N2 O6	258.0844	1.70e	1.76e	1.67e	3.09e	3.13e	2.78e	5.15e	5.17e	5.06e	2.06e	1.86e	1.68e

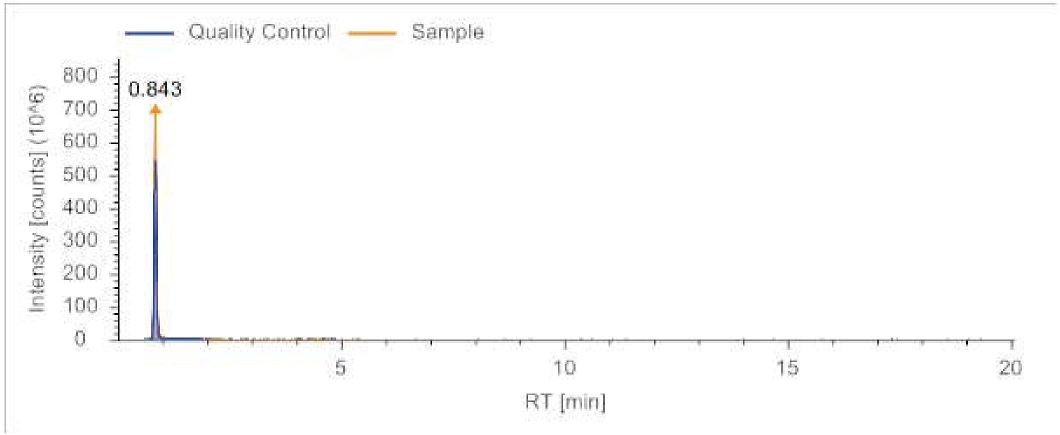

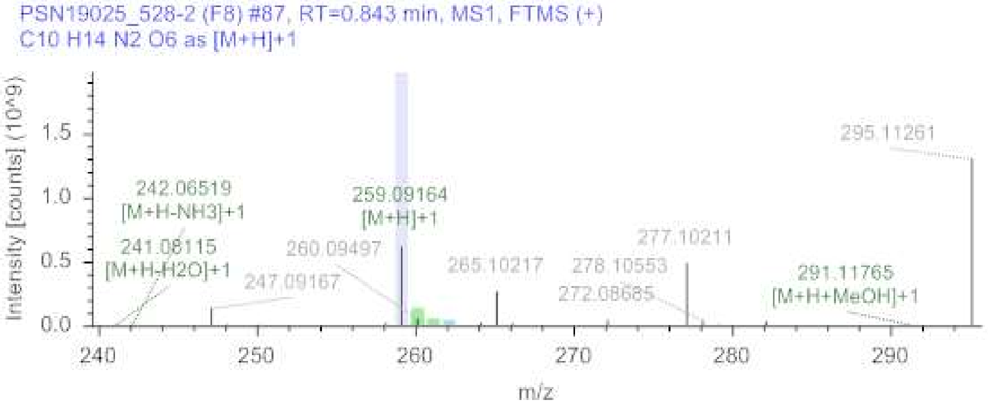

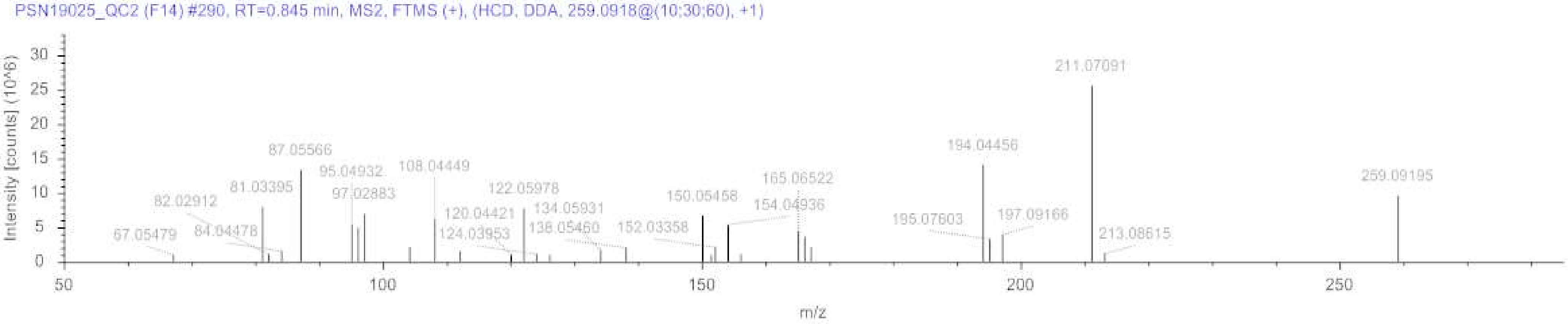

© Reported with Compound Discoverer 3.2

Compounds

Structure	Name	RT [min]	Formula	Molecular Weight	Group Areas											
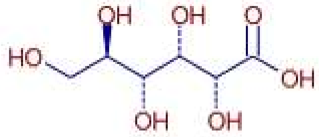	Gluconic acid	0.92	C6 H12 O7	196.0603	5.77e	5.99e	5.01e	1.97e	5.14e	9.21e	5.95e	5.35e	1.42e	9.41e	9.03e	.05e1

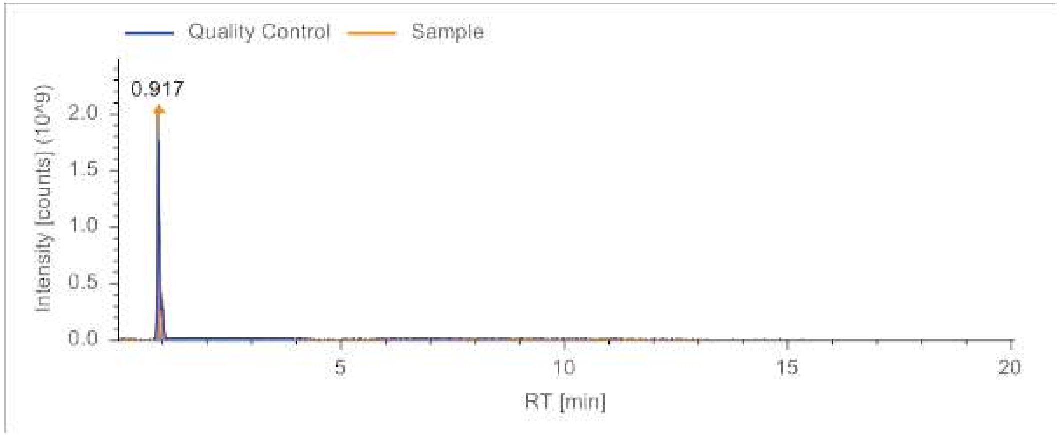

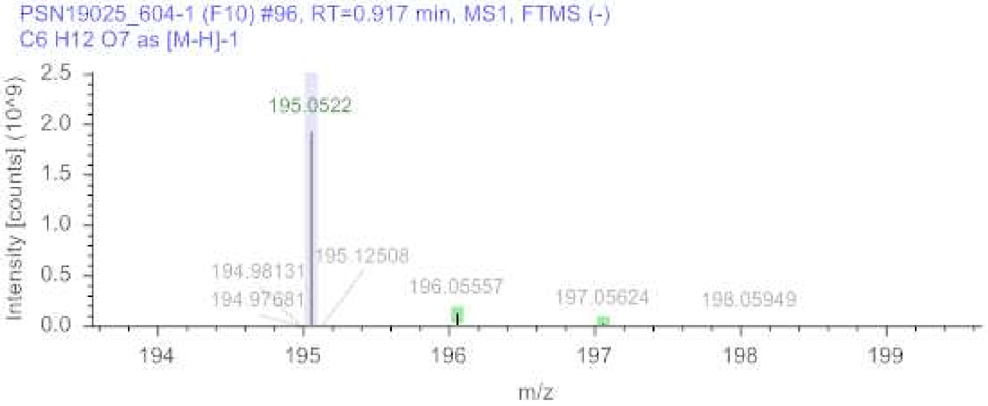

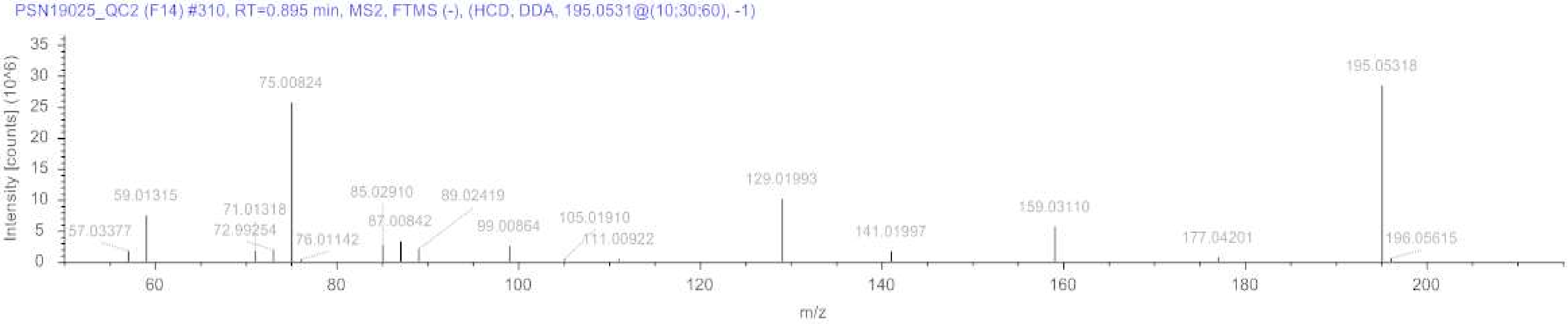

© Reported with Compound Discoverer 3.2

Compounds

Structure	Name	RT [min]	Formula	Molecular Weight	Group Areas											
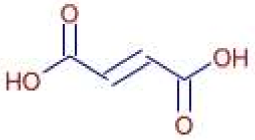	Fumaric acid	1.17	C4 H4 O4	134.0221	3.19e	3.70e	9.16e	1.20e	1.85e	1.18e	3.63e	3.62e	2.26e	.55e1	.46e1	.41e1

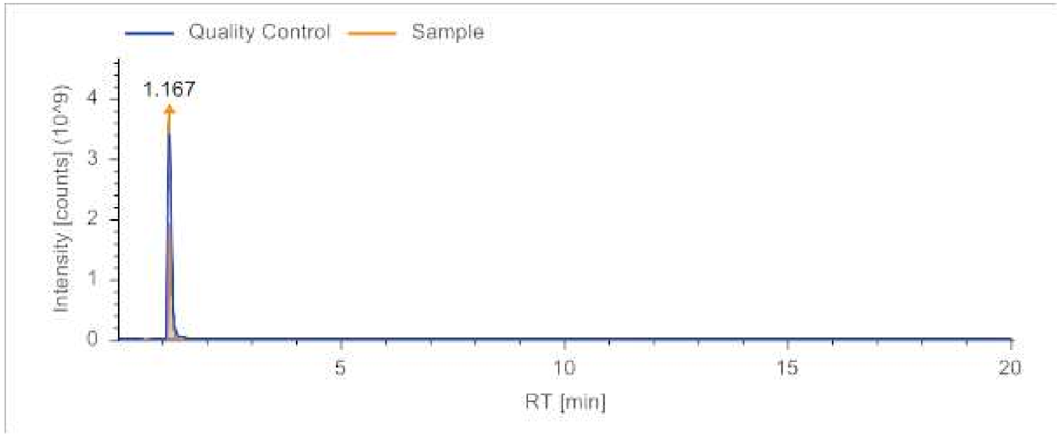

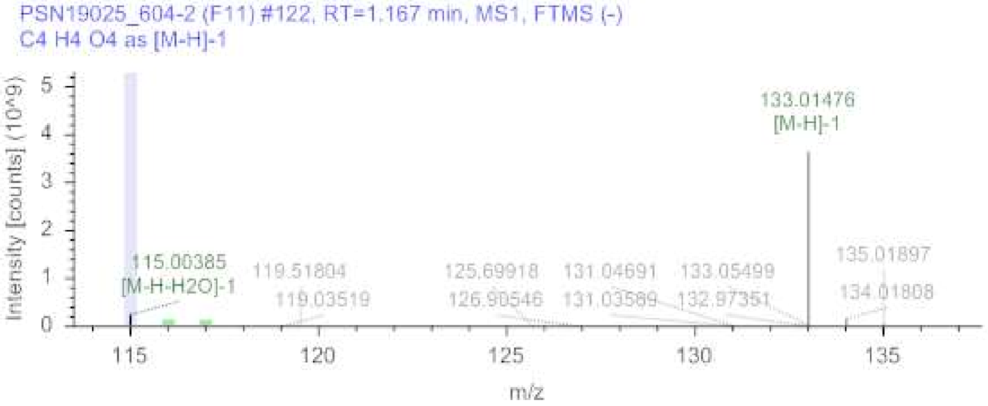

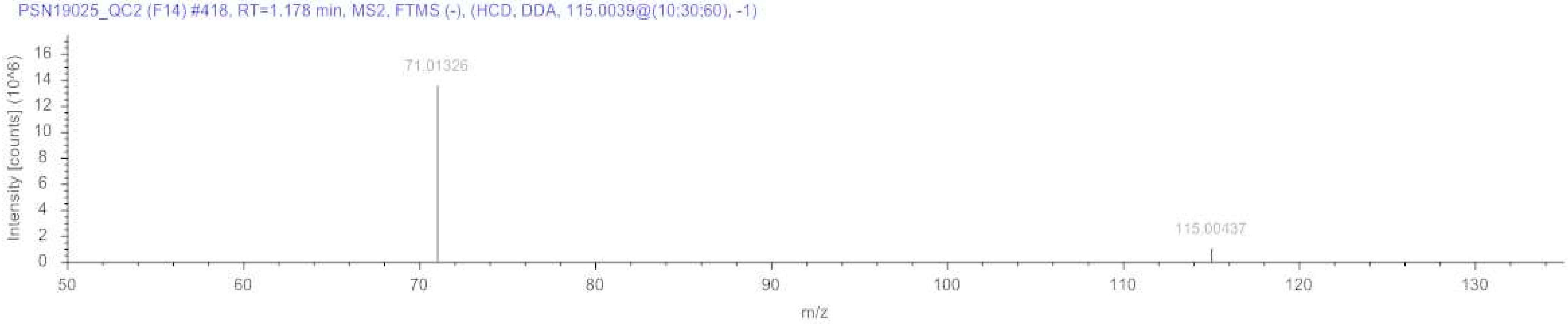

© Reported with Compound Discoverer 3.2

Compounds

Structure	Name	RT [min]	Formula	Molecular Weight	Group Areas											
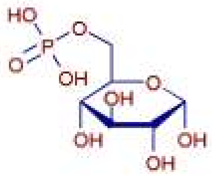	D-Glucose 6-phosphate	1.53	C6 H13 O9 P	260.0335	1.46e9	1.62e9	1.58e9	7.46e[illegible]	7.14e[illegible]	5.16e[illegible]	1.10e[illegible]	1.00e[illegible]	9.89e[illegible]	2.08e9	2.18e9	1.67e[illegible]

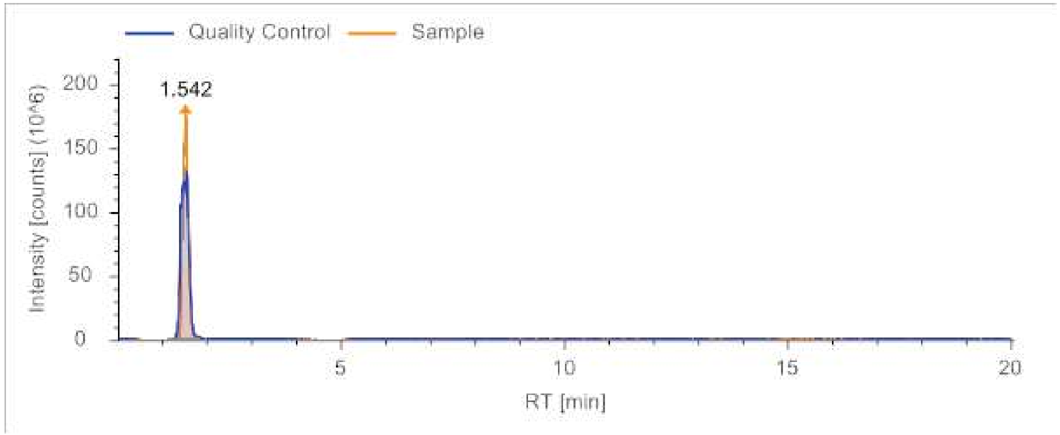

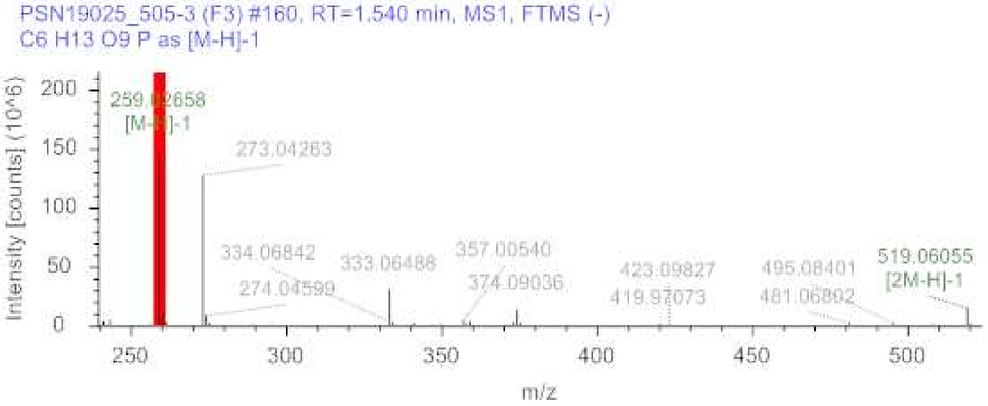

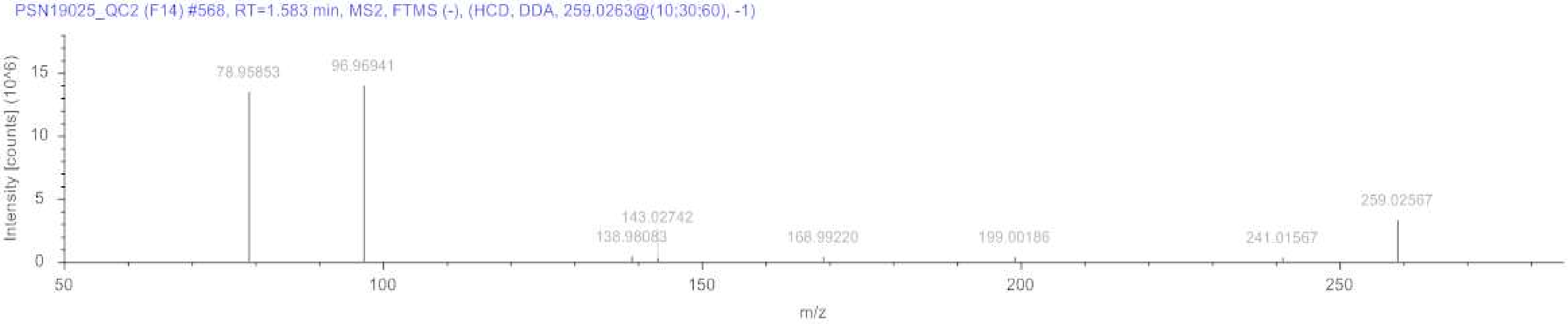

© Reported with Compound Discoverer 3.2

Compounds

Structure	Name	RT [min]	Formula	Molecular Weight	Group Areas
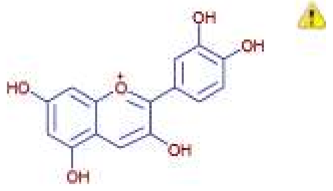	Cyanidin	4.48	C15 H10 O6	286.0467	2.10e 2.16e 2.22e 3.44e 3.76e 3.10e 3.08e 2.12e 1.94e 1.55e 1.63e 1.63e

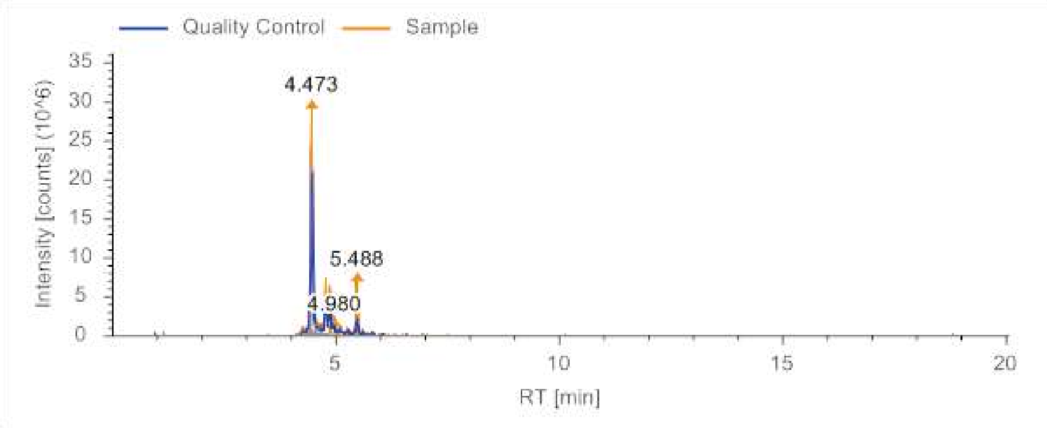

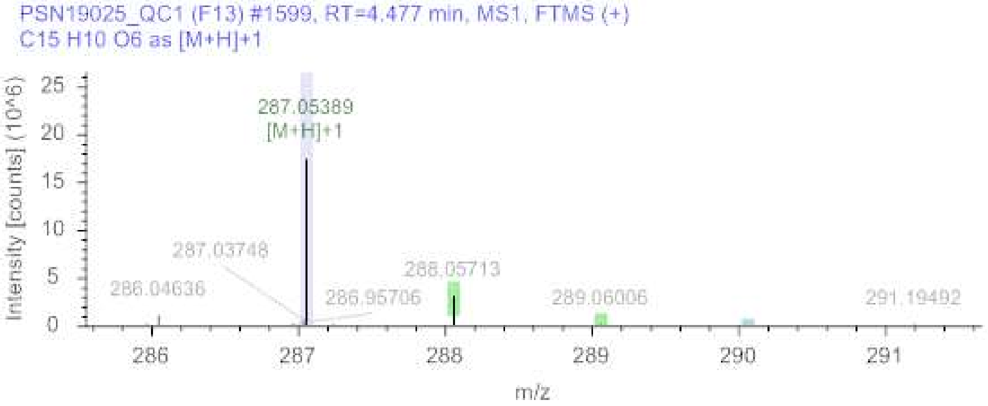

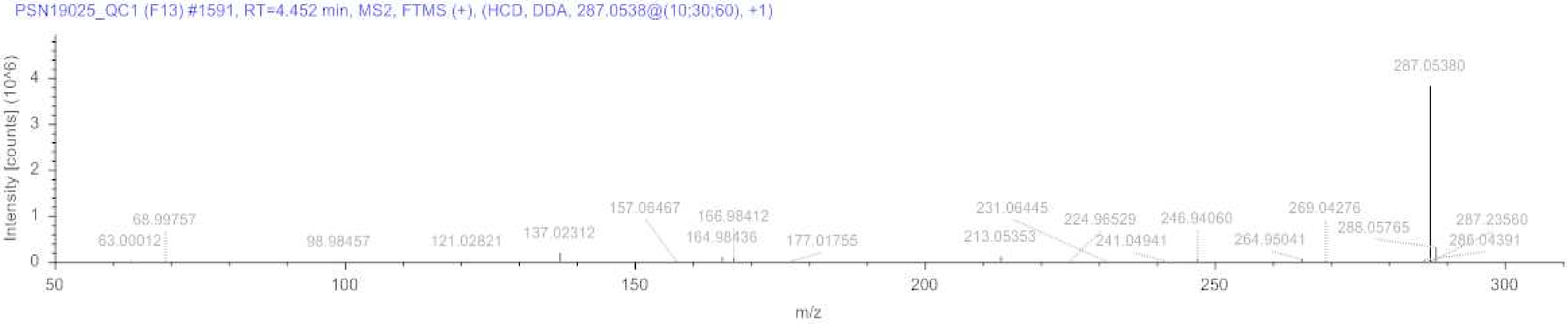

© Reported with Compound Discoverer 3.2

Compounds

Structure	Name	RT [min]	Formula	Molecular Weight	Group Areas											
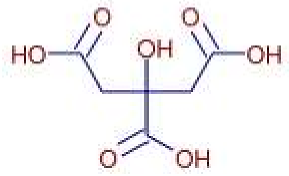	Citric acid	1.71	C6 H8 O7	192.0291	1.77e[illegible]	1.83e[illegible]	1.79e[illegible]	2.37e[illegible]	2.40e[illegible]	2.16e[illegible]	2.97e[illegible]	2.88e[illegible]	2.75e[illegible]	1.43e[illegible]	1.20e[illegible]	1.23e[illegible]

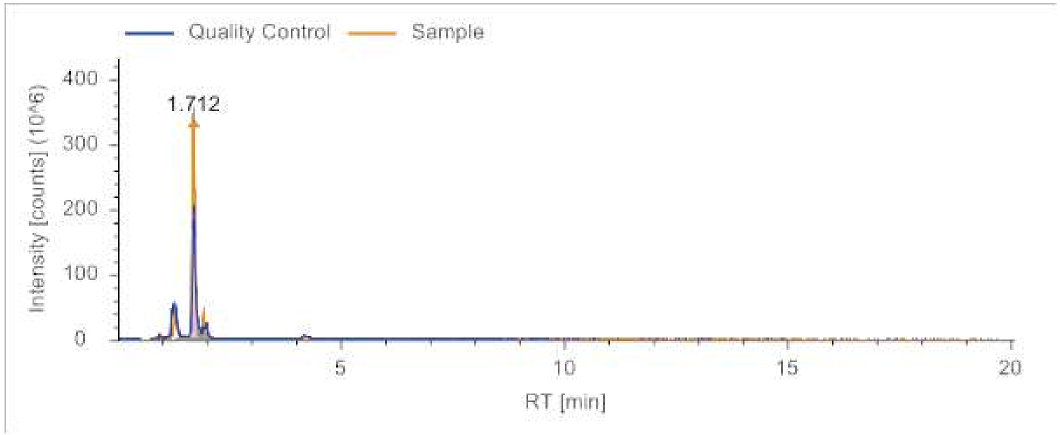

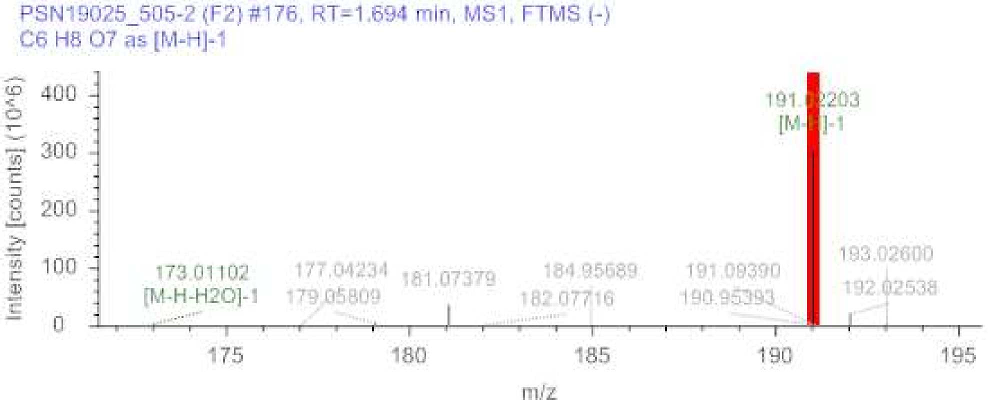

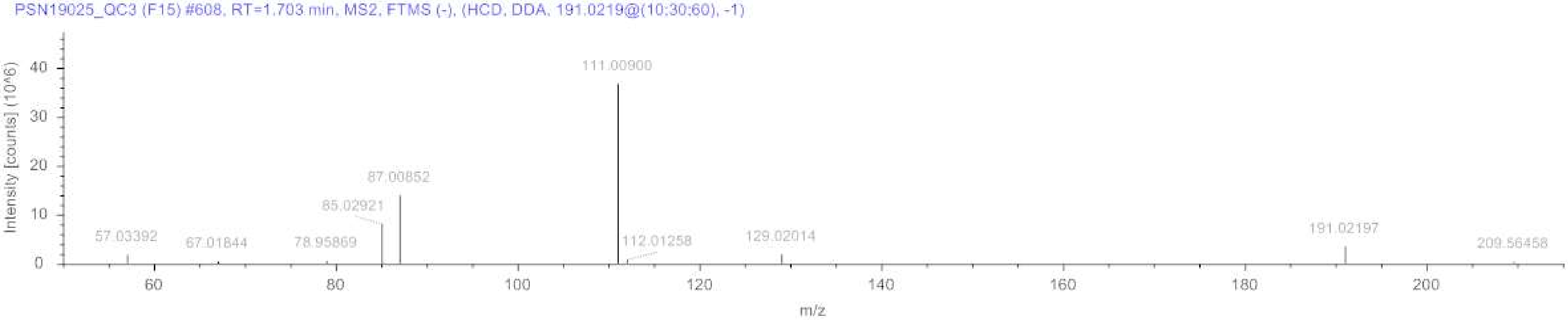

© Reported with Compound Discoverer 3.2

Structure	Name	RT [min]	Formula	Molecular Weight	Group Areas											
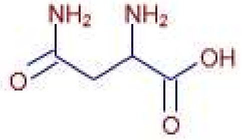	Asparagine	0.81	C4 H8 N2 O3	132.0540	7.23e	5.80e	5.06e	3.45e	.17e1	.02e1	1.57e	1.24e	3.84e	.09e1	3.02e	9.76e

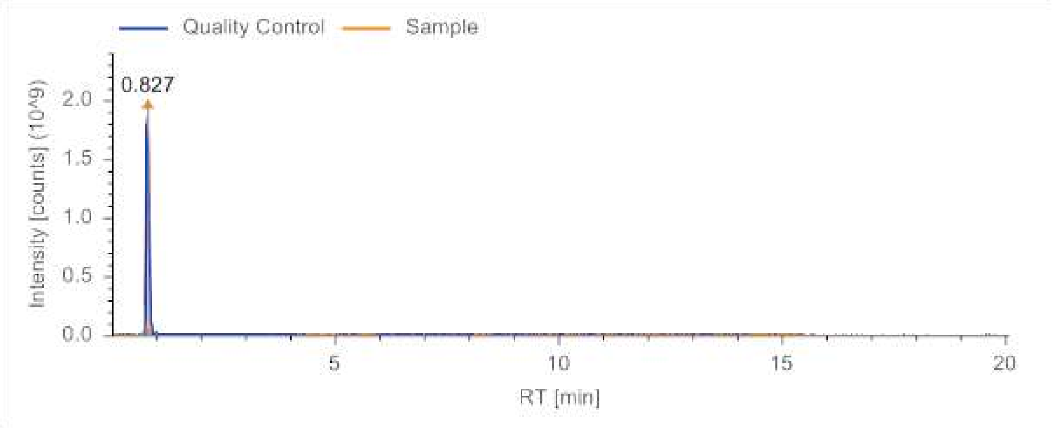

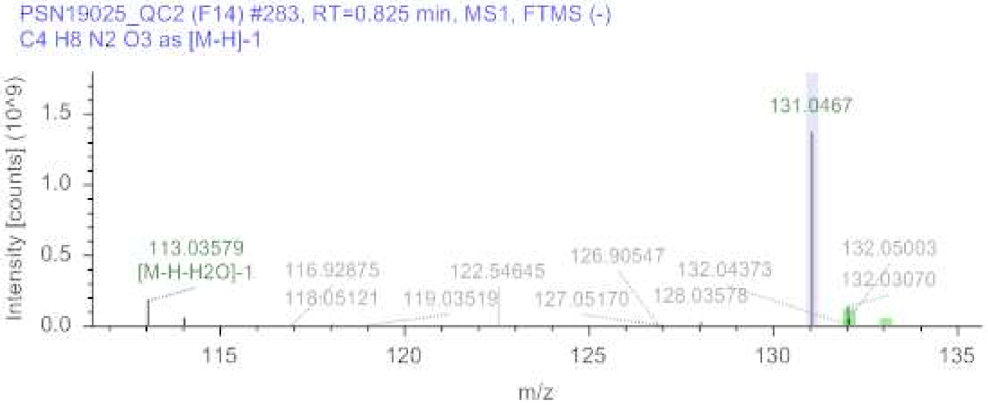

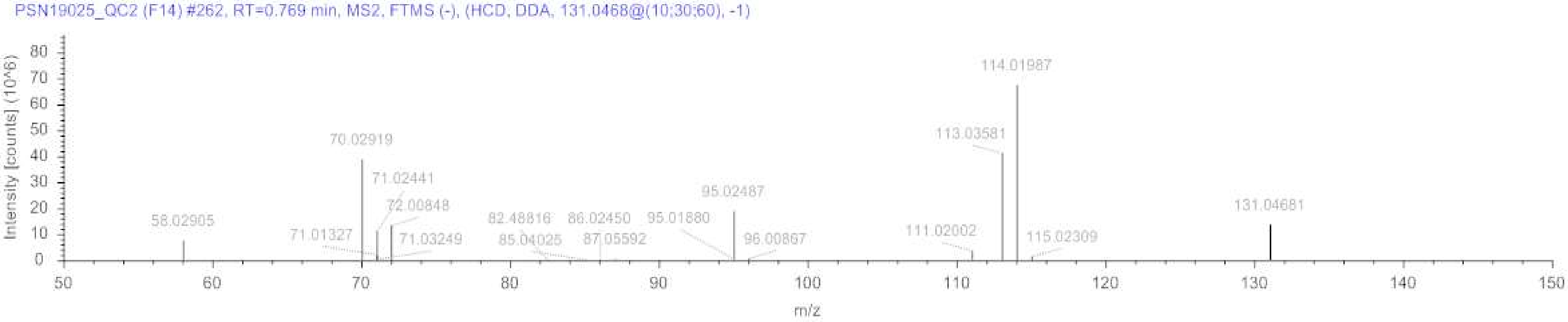

© Reported with Compound Discoverer 3.2

Compounds

Structure	Name	RT [min]	Formula	Molecular Weight	Group Areas											
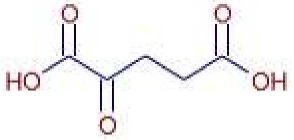	2-Oxoglutaric acid	2.01	C5 H6 O5	146.0224	2.09e	1.87e	1.94e	1.04e	1.15e	1.42e	1.53e	1.25e	2.08e	9.62e	9.20e	9.96e

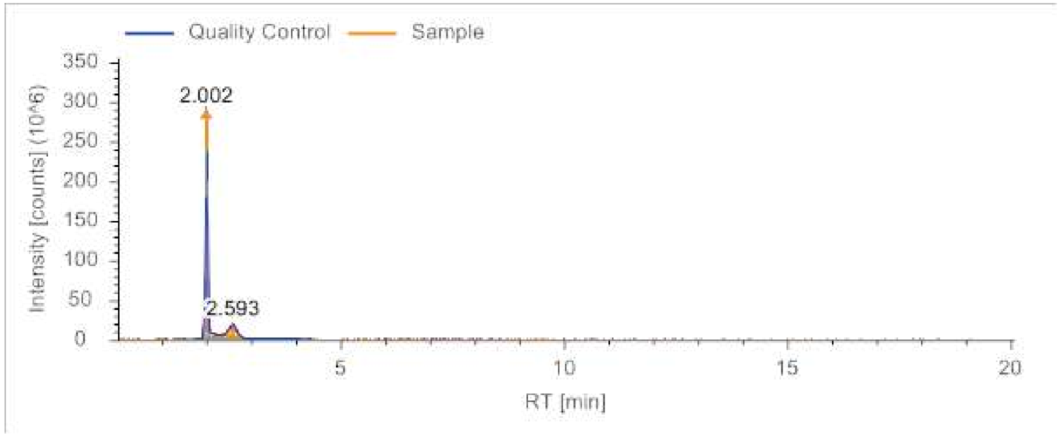

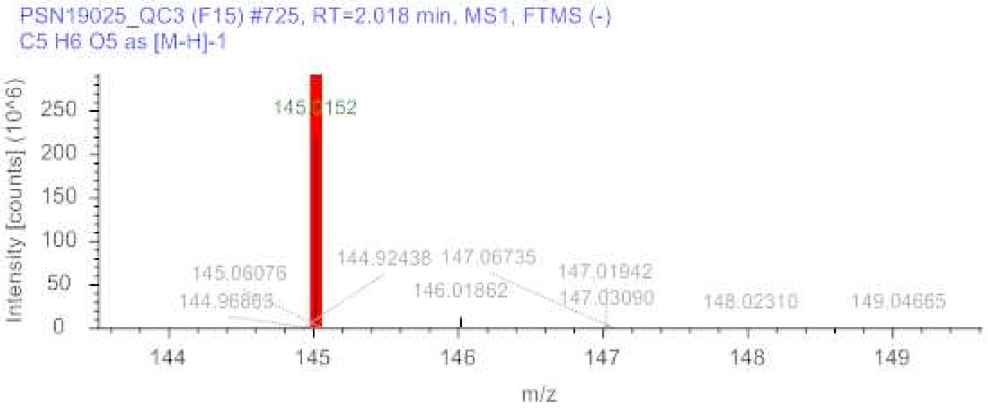

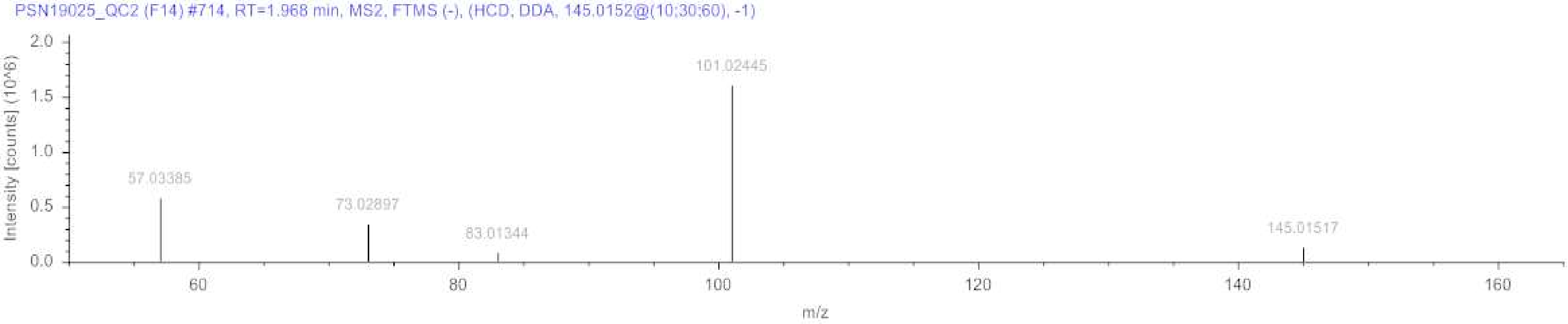

© Reported with Compound Discoverer 3.2
